# Both soluble and cell surface CD137 expressed by Foxp3^+^ CD4 T cells restrain autoimmune diabetes

**DOI:** 10.1084/jem.20252647

**Published:** 2026-07-07

**Authors:** Rabia Nabi, Chien-Wei Lin, Yu Wang, Ashley E. Ciecko, Bardees M. Foda, Yushu Wang, Amber Drewek, Scott M. Lieberman, William M. Ridgway, Yi-Guang Chen

**Affiliations:** 1 Department of Pediatrics, Medical College of Wisconsin, Milwaukee, WI, USA; 2 Max McGee Research Center for Juvenile Diabetes, Medical College of Wisconsin, Milwaukee, WI, USA; 3 Division of Biostatistics, Data Science Institute, Medical College of Wisconsin, Milwaukee, WI, USA; 4 Department of Microbiology and Immunology, Medical College of Wisconsin, Milwaukee, WI, USA; 5 Department of Molecular Genetics and Enzymology, National Research Centre, Dokki, Egypt; 6 Stead Family Department of Pediatrics, Carver College of Medicine, University of Iowa, Iowa City, IA, USA; 7 Interdisciplinary Graduate Program in Immunology, University of Iowa, Iowa City, IA, USA; 8 Division of Rheumatology, Allergy and Clinical Immunology, University of California, Davis, Davis, CA, USA

## Abstract

CD137 is expressed in a subset of Foxp3^+^ regulatory CD4 T cells (Tregs), but its immunoregulatory role is not fully defined. Due to alternative splicing that removes the transmembrane domain–encoding exon, CD137 exists in both membrane and soluble forms. We investigated the function of CD137 in Foxp3^+^ Tregs using the NOD mouse model of type 1 diabetes (T1D). Foxp3^+^ Treg–specific deletion of CD137 reduced circulating soluble CD137 and accelerated T1D development, driven by heightened clonal expansion and differentiation of effector T cells in pancreatic islets. CD137 deficiency in Foxp3^+^ Tregs reduced their frequency in islets and impaired their differentiation toward a suppressive phenotype. Restoring soluble CD137 in Foxp3^+^ Tregs lacking its membrane form reduced islet T cell activation and mitigated T1D acceleration without altering the accumulation of suppressive Foxp3^+^ Tregs. Our results indicate that both soluble and membrane forms of CD137 expressed by Foxp3^+^ Tregs are critical for immunoregulation, and they independently restrain T1D development.

## Teaser

Due to alternative splicing that removes the transmembrane domain–encoding exon, CD137 exists in both soluble and membrane forms. This study indicates that both forms of CD137 expressed by Foxp3^+^ CD4 T cells are critical for immunoregulation, and they independently restrain T1D development.

## Graphical Abstract

**Figure:**
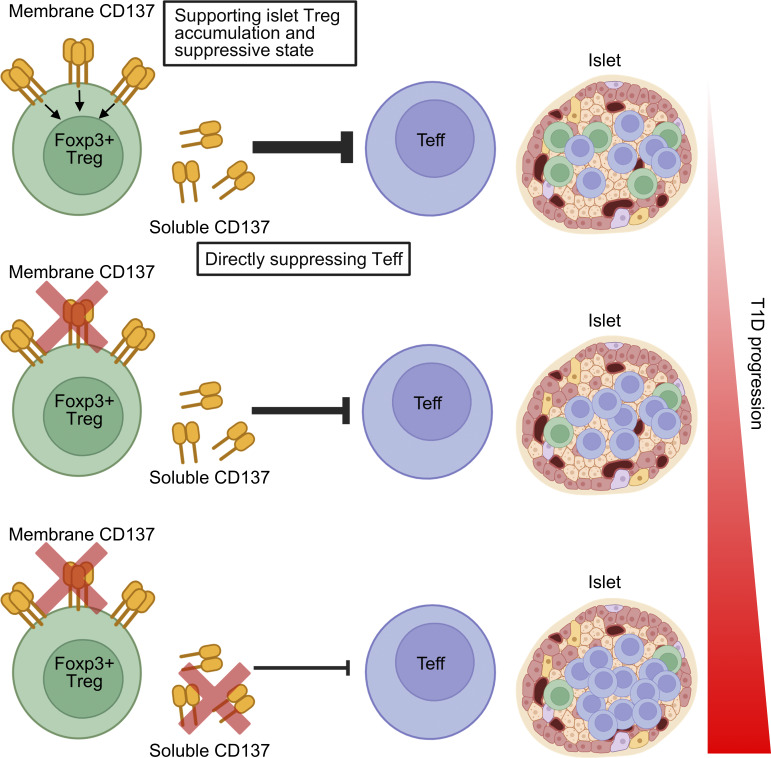

## Introduction

CD137 (4-1BB), encoded by *Tnfrsf9*, belongs to the tumor necrosis factor receptor superfamily (TNFRSF) whose members regulate various functions of immune cells (Watts et al., 2025). While CD137 expression is not restricted to T cells, it is a well-known costimulatory molecule upregulated on activated T cells and interacts with CD137 ligand (CD137L) expressed on APCs (Goodwin et al., 1993; Kwon and Weissman, 1989; Shuford et al., 1997). CD137 signaling in CD8 T cells promotes their survival and metabolic fitness and controls their exhaustion during chronic antigen stimulation (Choi et al., 2017; Lee et al., 2002; Menk et al., 2018; Pichler et al., 2023; Sabbagh et al., 2008; Teijeira et al., 2018; Vezys et al., 2011). CD137 is expressed on a subset of Foxp3^+^ regulatory CD4 T cells (Tregs) (Irie et al., 2007; Marson et al., 2007; Zheng et al., 2004). However, the role of CD137 in Foxp3^+^ Tregs is not well defined. Previous studies testing the role of CD137 in Tregs using agonistic anti-CD137 antibodies have led to inconsistent results (Akhmetzyanova et al., 2016; Choi et al., 2004; Elpek et al., 2007; Imianowski et al., 2024; Kim et al., 2012; Zhang et al., 2007; Zheng et al., 2004). However, anti-CD137 stimulation does not reflect physiological conditions. CD137 is also expressed as a soluble protein by Tregs due to alternative splicing that excludes the transmembrane domain–encoding exon (Kachapati et al., 2012; Setareh et al., 1995), and the effect of anti-CD137 on the function of soluble CD137 is not known.

We conducted studies in the past to uncover the functions of CD137 using the NOD mouse model of type 1 diabetes (T1D). NOD mice congenic for the C57BL/10 (B10)-derived *Idd9.3* region on chromosome 4 have increased CD137^+^ Foxp3^+^ Tregs and serum soluble CD137, and are more resistant to T1D when compared to standard NOD mice (Forsberg et al., 2019; Kachapati et al., 2012; Lyons et al., 2000). The different levels of CD137^+^ Foxp3^+^ Tregs and circulating soluble CD137 observed in NOD and NOD.B10-*Idd9.3* mice are controlled by the respective NOD and B10 *Tnfrsf9* alleles within the *Idd9.3* region (Forsberg et al., 2019). Soluble CD137 directly suppresses effector CD4 and CD8 T cells *in vitro* in an APC-independent but CD137L-dependent fashion, and it prevents spontaneous T1D, and ameliorates acute T1D, when injected into NOD mice (Itoh et al., 2019; Kachapati et al., 2013). T cells transiently upregulate *Tnfsf9* (CD137L) upon activation, and soluble CD137 inhibits their mTORC1 signaling pathway possibly through CD137L reverse signaling (Itoh et al., 2019). CD137^+^ Foxp3^+^ Tregs are more suppressive than the CD137^−^ counterpart *in vitro* (Kachapati et al., 2012). Interestingly, CD137 expressed in CD4 and CD8 T cells, respectively, inhibits and promotes T1D in NOD mice (Forsberg et al., 2017). This is because CD137-CD137L interaction is important for the survival and expansion of β-cell autoreactive CD8 T cells (Foda et al., 2020; Forsberg et al., 2017). The T1D protective activity of CD137 in CD4 T cells could be due to its expression in Foxp3^+^ Tregs as they produce more soluble CD137 than conventional T cells in culture (Kachapati et al., 2012). Collectively, our results support but have not directly proved that Foxp3^+^ Treg–derived soluble CD137 is immunoregulatory and inhibits T1D development *in vivo*.

As in mice, human *TNFRSF9* also undergoes alternative splicing to produce both membrane and soluble forms of CD137 (Michel et al., 1998; Rojas et al., 2025). Upon *in vitro* activation, human Foxp3^+^ CD4 Tregs express more membrane and soluble CD137 compared with conventional CD4 T cells (Rojas et al., 2025). Recombinant human Fc-soluble CD137 suppresses IFNγ production and proliferation of human CD4 and CD8 T cells in an APC-independent manner (Rojas et al., 2025). Significantly, lower levels of serum soluble CD137 have been reported in T1D patients compared with age-matched controls (Itoh et al., 2019). Although soluble CD137 is clearly important in regulating mouse T1D and available evidence supports its relevance in human T1D, our understanding of the role of CD137 in Foxp3^+^ Tregs is incomplete. First, the *in vivo* evidence for the contribution of Foxp3^+^ Treg–derived soluble CD137 to T1D suppression is not definitive. It is also not known whether cell surface CD137 plays a unique role in Foxp3^+^ Tregs, since other TNFRSF proteins can redundantly induce similar downstream signaling (Lubrano di Ricco et al., 2020; Vasanthakumar et al., 2017). Second, *Tnfrsf9* global knockout does not lead to systemic inflammation but conversely suppresses T1D in NOD mice (Chen et al., 2014). This could mean that CD137 expression in Foxp3^+^ Tregs is not essential for controlling autoreactive T cells; however, this interpretation is confounded by the concurrent lack of CD137-mediated costimulatory function in CD8 T cells (Forsberg et al., 2017). Third, the effects of CD137 deficiency in Foxp3^+^ Tregs on other immune cells are completely unknown. Here, we constructed Foxp3^+^ Treg–specific *Tnfrsf9* knockout mice that lacked either all CD137 molecules (membrane and soluble) or only the membrane form to address these questions.

## Results

### Foxp3^+^ Treg–specific deletion of CD137 accelerates T1D development

**Figure S1.**
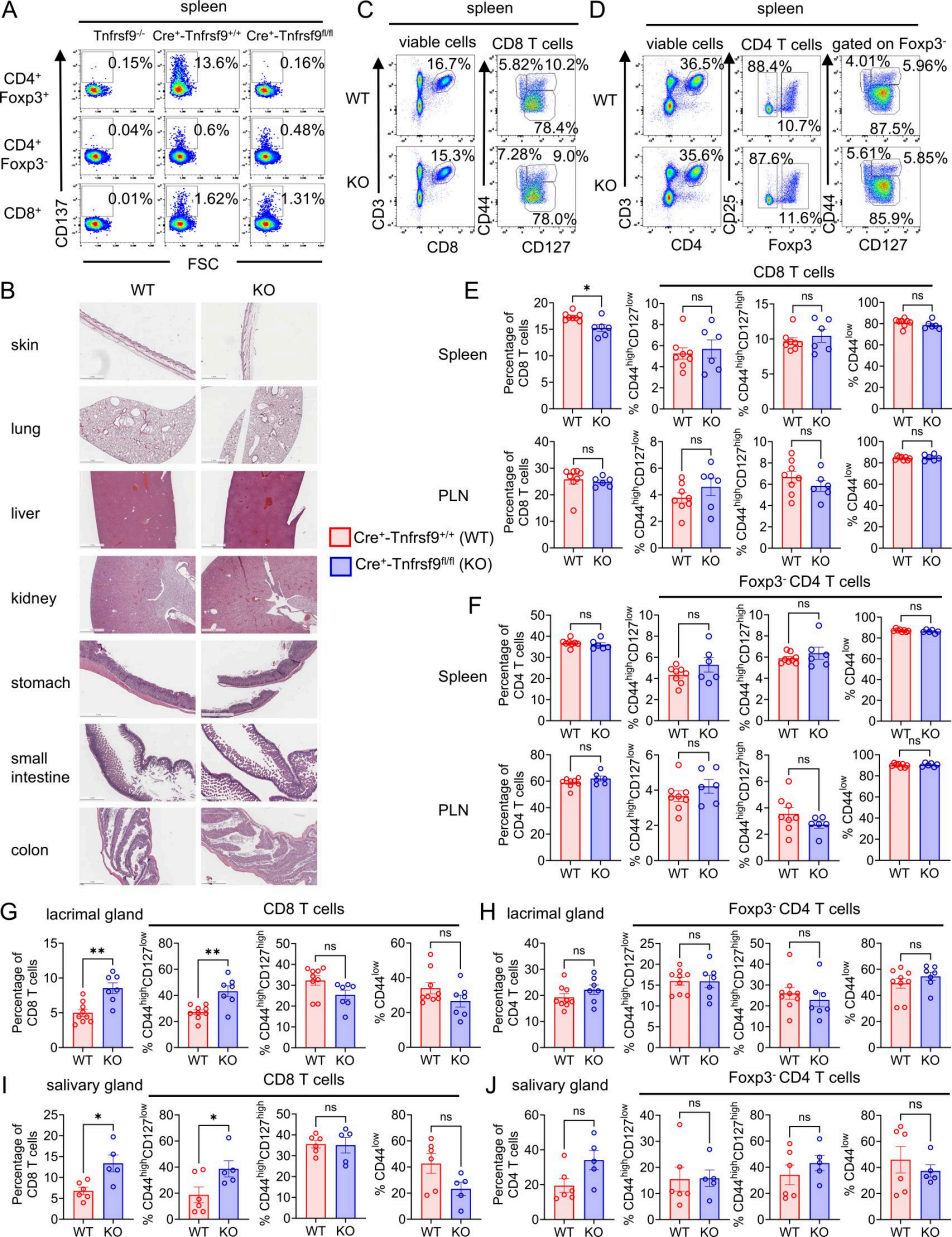
**Characterization of Cre** ^**+**^ **-*Tnfrsf9*** ^**+/+**^ **and Cre** ^**+**^ **-*Tnfrsf9*** ^**fl/fl**^ **mice, related to Fig. 1. (A)** CD137 expression on splenic Foxp3^+^ and Foxp3^−^ CD4 T cells and CD8 T cells of *Cre*^+^-*Tnfrsf9*^+/+^ and *Cre*^+^-*Tnfrsf9*^fl/fl^ mice. NOD.*Tnfrsf9*^−/−^ cells were used as the negative control. Representative flow cytometry profiles of three mice per genotype are shown. **(B)** Representative histological images of indicated tissues of three prediabetic 10- to 11-wk-old female mice per genotype are shown. The scale bar is 1 mm. Similar results were observed in three age-matched male mice per genotype. **(C and D)** Representative flow cytometry plots of splenic CD8 (C) and CD4 (D) T cells in 6- to 8-wk-old *Cre*^+^-*Tnfrsf9*^+/+^ (WT) and *Cre*^+^-*Tnfrsf9*^fl/fl^ (KO) females. The CD3 by CD4 and CD25 by Foxp3 plots of the KO spleen are also shown in Fig. 1 A. **(E)** Percentages of splenic and PLN total CD8 T cells in 6- to 8-wk-old *Cre*^+^-*Tnfrsf9*^+/+^ (WT) and *Cre*^+^-*Tnfrsf9*^fl/fl^ (KO) females and their activation status determined by the expression levels of CD44 and CD127. Summarized results from three experiments are shown. *P < 0.05 by an unpaired *t* test. ns: not significant. **(F)** Percentages of splenic and PLN total CD4 T cells and the activation status of Foxp3^−^ CD4 T cells in 6- to 8-wk-old *Cre*^+^-*Tnfrsf9*^+/+^ (WT) and *Cre*^+^-*Tnfrsf9*^fl/fl^ (KO) females determined by the levels of CD44 and CD127 expression. Summarized results from three experiments are shown. ns: not significant by an unpaired *t* test. **(G and H)** Percentages of total CD8 (G) and CD4 (H) T cells and their activation status in lacrimal glands of 9- to 11-wk-old *Cre*^+^-*Tnfrsf9*^+/+^ (WT) and *Cre*^+^-*Tnfrsf9*^fl/fl^ (KO) male mice. Results are summarized from three experiments. **P < 0.005 by an unpaired *t* test. ns: not significant. **(I and J)** Percentages of total CD8 (I) and CD4 (J) T cells and their activation status in salivary glands of 9- to 10-wk-old *Cre*^+^-*Tnfrsf9*^+/+^ (WT) and *Cre*^+^-*Tnfrsf9*^fl/fl^ (KO) female mice. Results are summarized from two experiments. *P < 0.05 by an unpaired *t* test. ns: not significant.

**Figure 1.**
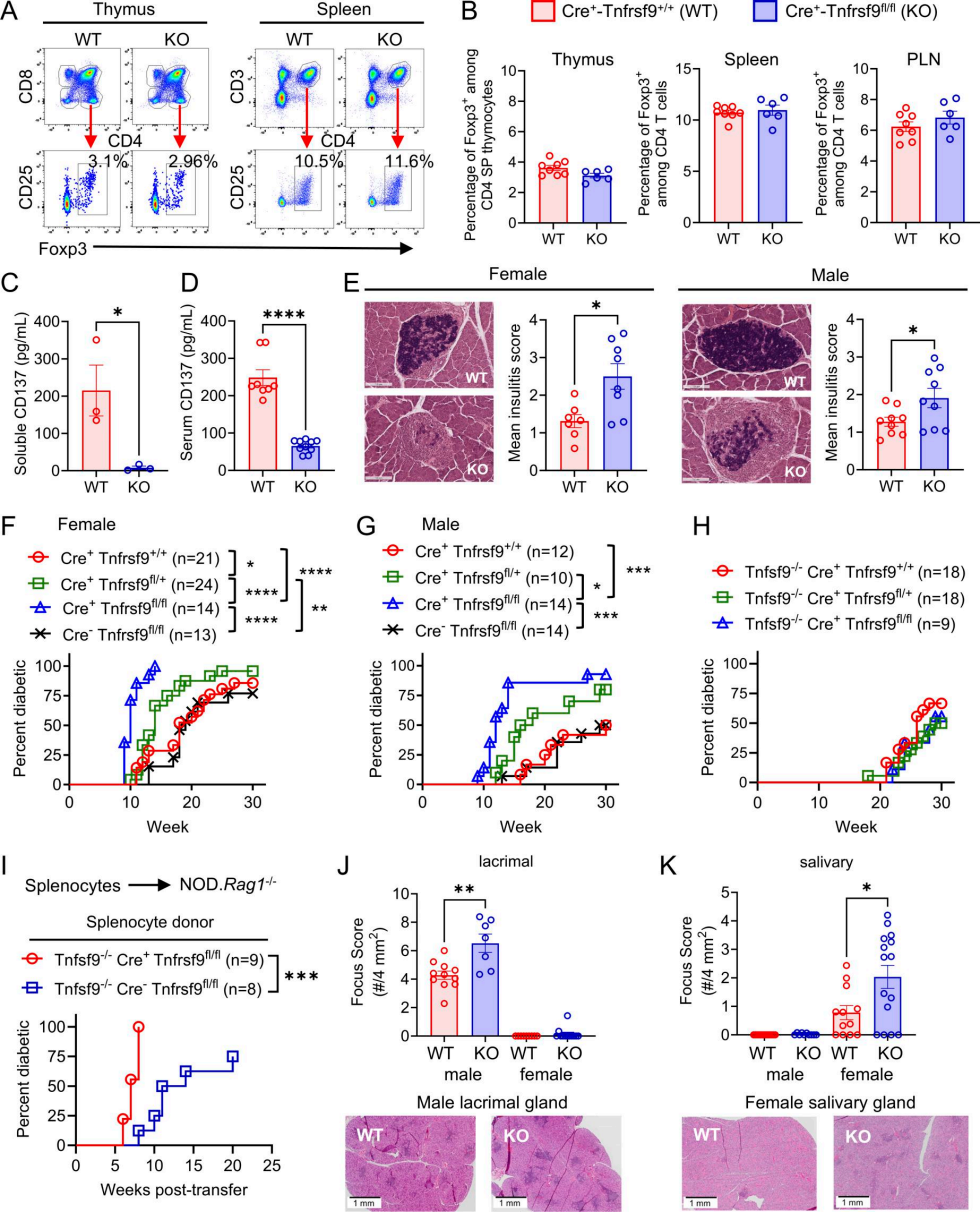
**CD137 deletion in Foxp3** ^**+**^ **Tregs leads to exacerbated diabetes development and Sjögren’s disease. (A and B)** Percentages of Foxp3^+^ Tregs in the thymus, spleen, and PLN of 6- to 8-wk-old *Cre*^+^-*Tnfrsf9*^+/+^ (WT) and *Cre*^+^-*Tnfrsf9*^fl/fl^ (KO) female mice. **(A)** Representative flow cytometry plots. The plots of the KO spleen are also shown in Fig. S1 D. **(B)** Summarized results from three experiments. **(C)** Soluble CD137 produced by cultured *Cre*^+^-*Tnfrsf9*^+/+^ and *Cre*^+^-*Tnfrsf9*^fl/fl^ splenic Foxp3^+^ Tregs (*n* = 3). *P < 0.05 by an unpaired *t* test. **(D)** Circulating soluble CD137 in 7- to 9-wk-old *Cre*^+^-*Tnfrsf9*^+/+^ and *Cre*^+^-*Tnfrsf9*^fl/fl^ males. ****P < 0.0001 by an unpaired *t* test. **(E)** Insulitis in 8- to 10-wk-old prediabetic *Cre*^+^-*Tnfrsf9*^fl/fl^ (KO) and *Cre*^+^-*Tnfrsf9*^+/+^ (WT) mice. Representative islet histology images and summarized mean insulitis scores are shown. The scale bar is 100 µm. *P < 0.05 by an unpaired *t* test. **(F and G)** T1D incidence of *Cre*^+^-*Tnfrsf9*^+/+^, *Cre*^+^-*Tnfrsf9*^fl/+^, *Cre*^+^-*Tnfrsf9*^fl/fl^, and *Cre*^−^-*Tnfrsf9*^fl/fl^ female (F) and male (G) littermates. *P < 0.05; **P < 0.005; ***P < 0.0005; ****P < 0.0001 by a log-rank test. **(H)** T1D incidence of CD137L-deficient (*Tnfsf9*^−/−^) *Cre*^+^-*Tnfrsf9*^+/+^, *Cre*^+^-*Tnfrsf9*^fl/+^, and *Cre*^+^-*Tnfrsf9*^fl/fl^ female littermates. **(I)** T1D incidence of NOD.*Rag1*^−/−^ females receiving CD137L-deficient (*Tnfsf9*^−/−^) *Cre*^+^-*Tnfrsf9*^fl/fl^ or *Cre*^−^-*Tnfrsf9*^*f*l/fl^ littermate splenocytes, combined from two transfer experiments. ***P < 0.0005 by a log-rank test. **(J and K)** Quantification of inflammation in lacrimal (J) and salivary (K) glands from 8- to 10-wk-old prediabetic *Cre*^+^-*Tnfrsf9*^+/+^ (WT) and *Cre*^+^-*Tnfrsf9*^fl/fl^ (KO) mice. Representative histology images of male lacrimal and female salivary glands and summarized focus scores are shown. The scale bar is 1 mm. *P < 0.05; **P < 0.005 by an unpaired *t* test.

To address the role of CD137 in Foxp3^+^ Treg function *in vivo*, we first generated NOD mice carrying a floxed *Tnfrsf9* allele (designated NOD.*Tnfrsf9*^fl/fl^). We crossed the NOD.*Tnfrsf9*^fl/fl^ stock to the NOD.*Foxp3*-*Cre* strain (Zhou et al., 2008) (hereafter *Cre*^+^-*Tnfrsf9*^+/+^) to generate Treg-specific *Tnfrsf9* knockout (designated *Cre*^+^-*Tnfrsf9*^fl/fl^). Deletion of CD137 was confined to Foxp3^+^ Tregs, and its expression in conventional CD4 and CD8 T cells was not significantly impacted (Fig. S1 A). *Cre*^+^-*Tnfrsf9*^fl/fl^ and *Cre*^+^-*Tnfrsf9*^+/+^ mice had similar levels of Foxp3^+^ Tregs in the thymus, spleen, and pancreatic lymph node (PLN) (Fig. 1, A and B). Thus, CD137 signaling is not required for the homeostasis of Foxp3^+^ Tregs in lymphoid tissues. Foxp3^+^ Tregs secrete more soluble CD137 than conventional T cells *in vitro* (Rojas et al., 2022), but it is not known whether Foxp3^+^ Tregs are the main soluble CD137 producers *in vivo*. We first confirmed that Foxp3^+^ Tregs isolated from *Cre*^+^-*Tnfrsf9*^fl/fl^ mice produced negligible soluble CD137 in culture (Fig. 1 C). Remarkably, serum soluble CD137 was reduced by 70% in *Cre*^+^-*Tnfrsf9*^fl/fl^ mice when compared to the *Cre*^+^-*Tnfrsf9*^+/+^ control, demonstrating that Foxp3^+^ Tregs are the major source of soluble CD137 *in vivo* (Fig. 1 D).

To determine the impact of CD137 deficiency in Foxp3^+^ Tregs on β-cell autoimmunity, we compared insulitis in 8- to 10-wk-old prediabetic *Cre*^+^-*Tnfrsf9*^+/+^ and *Cre*^+^-*Tnfrsf9*^fl/fl^ mice. Histological examination revealed that *Cre*^+^-*Tnfrsf9*^fl/fl^ mice had higher levels of insulitis in both sexes (Fig. 1 E). We also monitored littermates of *Cre*^+^-*Tnfrsf9*^+/+^, *Cre*^+^-*Tnfrsf9*^fl/+^, *Cre*^+^-*Tnfrsf9*^fl/fl^, and *Cre*^−^-*Tnfrsf9*^fl/fl^ for diabetes development. Mice with Treg-specific deletion of CD137 (*Cre*^+^-*Tnfrsf9*^fl/fl^) developed diabetes more rapidly than the wild-type littermates (*Cre*^+^-*Tnfrsf9*^+/+^ and *Cre*^−^-*Tnfrsf9*^fl/fl^) in both females and males (Fig. 1, F and G). Heterozygous mice (*Cre*^+^-*Tnfrsf9*^fl/+^) showed an intermediate diabetes incidence in both sexes (Fig. 1, F and G). Accelerated diabetes onset observed in *Cre*^+^-*Tnfrsf9*^fl/fl^ females was completely abolished when they were also globally deficient in CD137L (*Tnfsf9*^−/−^) (Fig. 1 H). Of note, CD137L deficiency in *Cre*^+^-*Tnfrsf9*^fl/fl^ females delayed T1D onset when compared to CD137L-sufficient *Cre*^+^-*Tnfrsf9*^+/+^ and *Cre*^−^-*Tnfrsf9*^fl/fl^ wild-type control mice (although they were not concurrently monitored, compare Fig. 1, F and H). This is because CD137L expressed by myeloid APCs is critical for the expansion and survival of β-cell autoreactive CD8 T cells (Foda et al., 2020). Interestingly, upon transferring into CD137L-expressing NOD.*Rag1*^−/−^ recipients, *Tnfsf9*^−/−^*Cre*^+^-*Tnfrsf9*^fl/fl^ splenocytes (lacking CD137 in Foxp3^+^ Tregs, and CD137L globally) caused rapid diabetes onset when compared to the *Tnfsf9*^−/−^*Cre*^−^-*Tnfrsf9*^fl/fl^ littermate control cells (with intact CD137 expression in Foxp3^+^ Tregs) (Fig. 1 I). As T cells did not express CD137L in this splenocyte transfer experiment, CD137 expressed by Foxp3^+^ Tregs suppressed T1D by interacting with CD137L on recipient myeloid APCs. The collective results indicate that Foxp3^+^ Treg–derived CD137 (membrane and/or soluble form) exerts its immunosuppressive functions through interaction with CD137L.

### Limited impact of Foxp3^+^ Treg–specific CD137 deletion on systemic inflammation

In addition to T1D, NOD mice also spontaneously develop autoimmunity toward male lacrimal and female salivary glands and are an established mouse model of Sjögren’s disease (Park et al., 2015). To evaluate whether Treg-specific CD137 deletion has a broader impact on autoimmunity, we analyzed other tissues in *Cre*^+^-*Tnfrsf9*^+/+^ and *Cre*^+^-*Tnfrsf9*^fl/fl^ mice. In *Cre*^+^-*Tnfrsf9*^fl/fl^ mice, increased infiltration was observed in male lacrimal glands and female salivary glands (Fig. 1, J and K). In contrast, no significant inflammation was observed in female lacrimal or male salivary glands, and histological analysis did not reveal abnormal inflammation in skin, lung, liver, kidney, stomach, small intestine, or colon in either sex (Fig. S1 B). Next, we analyzed the activation status of CD8 and CD4 T cells in the spleen and PLN of *Cre*^+^-*Tnfrsf9*^+/+^ and *Cre*^+^-*Tnfrsf9*^fl/fl^ mice (Fig. S1, C–F). The frequency but not the absolute number (not shown) of splenic CD8 T cells was slightly reduced in *Cre*^+^-*Tnfrsf9*^fl/fl^ mice, while the proportion of CD4 T cells was similar between strains. We used CD44 and CD127 (IL-7 receptor) to define the activation status of T cells (effector: CD44^high^CD127^low^; memory: CD44^high^CD127^high^; and naïve: CD44^low^). Both CD8 and Foxp3^−^ CD4 T cells showed similar activation status in the spleen and PLN of *Cre*^+^-*Tnfrsf9*^+/+^ and *Cre*^+^-*Tnfrsf9*^fl/fl^ mice, despite increased autoimmune inflammation in pancreatic islets of the latter strain. We also analyzed the activation status of T cells in male lacrimal and female salivary glands (Fig. S1, G–J). Compared with the *Cre*^+^*-Tnfrsf9*^+/+^ wild-type control, the frequency of CD8 T cells among CD45.1^+^ cells was increased in *Cre*^+^*-Tnfrsf9*^fl/fl^ mice in both male lacrimal and female salivary glands (Fig. S1, G and I). In both glands, more CD8 T cells displayed an effector phenotype (CD44^high^CD127^low^) in *Cre*^+^*-Tnfrsf9*^fl/fl^ than in *Cre*^+^*-Tnfrsf9*^+/+^ mice, indicating that CD8 T cells were more activated in the former strain (Fig. S1, G and I). A trend of increased CD4 T cells was observed at both anatomical sites, but statistical significance was not reached (Fig. S1, H and J). The activation status of CD4 T cells in both male lacrimal and female salivary glands was comparable between *Cre*^+^*-Tnfrsf9*^fl/fl^ and *Cre*^+^*-Tnfrsf9*^+/+^ mice (Fig. S1, H and J). Collectively, it appears that Foxp3^+^ Treg CD137 expression is not required for preventing systemic inflammation, but it restrains inflammation in tissues with ongoing autoimmunity.

### Foxp3^+^ Treg–specific CD137 deficiency drives islet T cell activation

**Figure 2.**
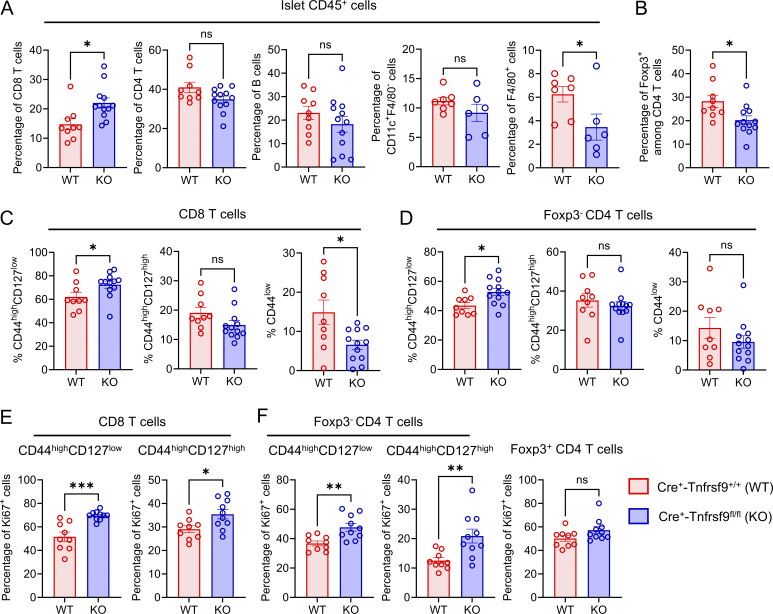
**CD137 deletion in Foxp3** ^**+**^ **Tregs causes enhanced T cell activation in pancreatic islets. (A)** Frequencies of T cells, B cells (CD19^+^), and myeloid cells in islets of 8- to 11-wk-old prediabetic *Cre*^+^-*Tnfrsf9*^+/+^ (WT) and *Cre*^+^-*Tnfrsf9*^fl/fl^ (KO) females. *P < 0.05 by an unpaired *t* test. ns: not significant. **(B)** Frequency of Foxp3^+^ Tregs in islets of 8- to 11-wk-old prediabetic *Cre*^+^-*Tnfrsf9*^+/+^ (WT) and *Cre*^+^-*Tnfrsf9*^fl/fl^ (KO) females. *P < 0.05 by an unpaired *t* test. **(C and D)** Frequencies of CD44^high^CD127^low^, CD44^high^CD127^high^, and CD44^low^ CD8 T cells (C) and Foxp3^−^ CD4 T cells (D) in islets of 8- to 11-wk-old prediabetic *Cre*^+^-*Tnfrsf9*^+/+^ (WT) and *Cre*^+^-*Tnfrsf9*^fl/fl^ (KO) females. *P < 0.05 by an unpaired *t* test. ns: not significant. **(E and F)** Frequencies of Ki67^+^ cells in the indicated subsets of CD8 (E) and CD4 (F) T cells in islets of 8- to 11-wk-old prediabetic *Cre*^+^-*Tnfrsf9*^+/+^ (WT) and *Cre*^+^-*Tnfrsf9*^fl/fl^ (KO) females. *P < 0.05, **P < 0.005; ***P < 0.0005 by an unpaired *t* test. ns: not significant. Results in A–F are summarized from three to five experiments.

As CD137 deletion in Foxp3^+^ Tregs led to accelerated T1D, we subsequently focused our analyses on islet-infiltrating cells. Compared with the *Cre*^+^-*Tnfrsf9*^+/+^ control, the frequencies of CD8 T cells and F4/80^+^ myeloid cells among CD45^+^ leukocytes were found respectively increased and decreased in the islets of *Cre*^+^-*Tnfrsf9*^fl/fl^ mice, while the levels of CD4 T cells, B cells, and CD11c^+^F4/80^−^ myeloid cells were similar between strains (Fig. 2 A). In addition, the percentage of Foxp3^+^ Tregs among islet-infiltrating CD4 T cells was reduced in *Cre*^+^-*Tnfrsf9*^fl/fl^ mice (Fig. 2 B). Based on the CD44 and CD127 expression patterns, there were proportionally more effector and less naïve CD8 T cells in *Cre*^+^-*Tnfrsf9*^fl/fl^ mice than in the *Cre*^+^-*Tnfrsf9*^+/+^ control (Fig. 2 C). Similarly, the effector population within Foxp3^−^ CD4 T cells was proportionally increased in *Cre*^+^-*Tnfrsf9*^fl/fl^ mice (Fig. 2 D). We previously showed that soluble CD137 suppressed T cell proliferation (Itoh et al., 2019; Kachapati et al., 2013). Consistently, Ki67 expression was significantly increased in effector and memory populations of both CD8 and Foxp3^−^ CD4 T cells in *Cre*^+^-*Tnfrsf9*^fl/fl^ mice (Fig. 2, E and F). In contrast, Ki67 expression in Foxp3^+^ Tregs was comparable between strains, suggesting that the reduced frequency of these cells in *Cre*^+^-*Tnfrsf9*^fl/fl^ mice was not due to decreased cell proliferation (Fig. 2 F).

**Figure S2.**
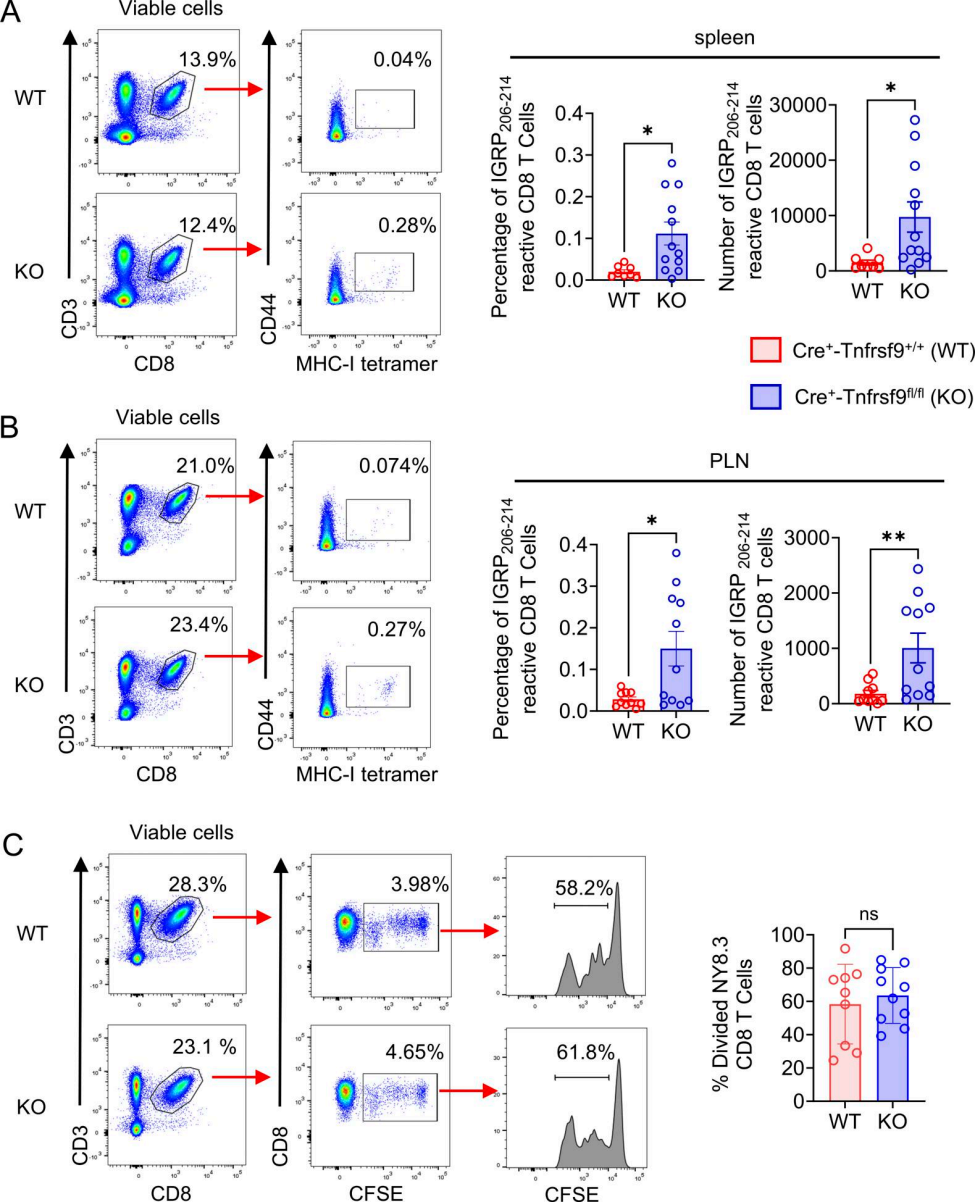
**Increased accumulation of IGRP**_**206-214**_ **-specific CD8 T cells in the spleen and PLN of Cre** ^**+**^ **-*Tnfrsf9*** ^**fl/fl**^ **mice, related to Fig. 2. (A and B)** Frequencies and absolute number of IGRP_206–214_-reactive CD8 T cells in the spleen (A) and PLN (B) of 7- to 9-wk-old Cre^+^-*Tnfrsf9*^+/+^ (WT) and Cre^+^-*Tnfrsf9*^fl/fl^ (KO) female mice. The representative flow cytometry profiles (left) and the summarized results (right) from four experiments are shown. *P < 0.05; **P < 0.01 by an unpaired *t* test. **(C)** Proliferation of adoptively transferred NY8.3 CD8 T cells in the PLNs of 7- to 9-wk-old Cre^+^-*Tnfrsf9*^+/+^ (WT) and Cre^+^-*Tnfrsf9*^fl/fl^ (KO) male mice. Splenocytes isolated from 7- to 9-wk-old NOD.*Rag1*^−/−^.*NY8.3* mice were labeled with CFSE and transferred into WT or KO mice. The gating strategy (left) and combined results (right) from five experiments are presented. ns: not significant by an unpaired *t* test.

In NOD mice, β-cell autoreactive CD8 T cells can emigrate from the islets and accumulate over time in lymphoid tissues (Chee et al., 2014). We asked whether the preferential expansion of islet-infiltrating CD8 T cells in islets of *Cre*^+^-*Tnfrsf9*^fl/fl^ mice is mirrored by an increase of β-cell autoreactive CD8 T cells in lymphoid tissues. The percentages and numbers of CD44^high^ IGRP_206-214_-reactive β-cell autoreactive CD8 T cells were significantly increased in the spleen and PLN of *Cre*^+^-*Tnfrsf9*^fl/fl^ mice compared with the *Cre*^+^-*Tnfrsf9*^+/+^ control (Fig. S2, A and B). This is not due to altered proliferation during the initial priming as adoptively transferred IGRP_206-214_-specific NY8.3 TCR transgenic CD8 T cells proliferated equally in the PLNs of *Cre*^+^-*Tnfrsf9*^fl/fl^ and *Cre*^+^-*Tnfrsf9*^+/+^ mice (Fig. S2 C). This further supports that CD137 expression in Foxp3^+^ Tregs is not required for regulating T cell activation in peripheral lymphoid tissues.

### CD137 deficiency in Foxp3^+^ Tregs alters their phenotype and drives islet effector T cell clonal expansion and terminal differentiation

**Figure S3.**
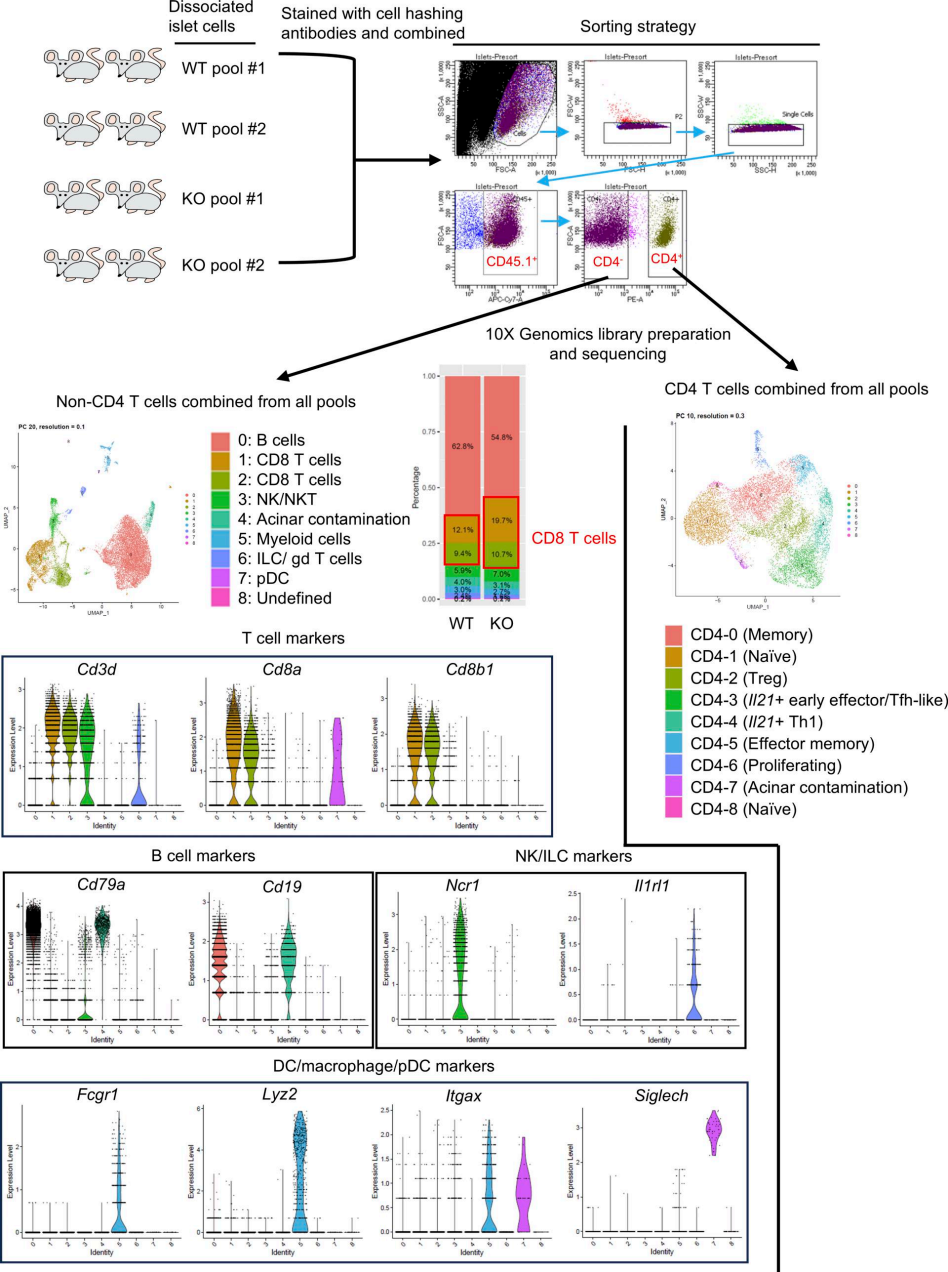
**Study design and cell sorting strategy for scRNA-seq analysis, related to Figs. 3, 4, and 5.** Two pools of islet cells combined from two Cre^+^-*Tnfrsf9*^+/+^ (WT) or Cre^+^-*Tnfrsf9*^fl/fl^ (KO) female mice were labeled with anti-CD45.1, anti-CD4, and a unique hashtag antibody for each cell pool. Stained cells were combined and sorted into CD45.1^+^ CD4^+^ T cells and non-CD4 T cells. Transcriptomic, V(D)J, and hashtag libraries were prepared for both cell populations and sequenced. Cell identities for unique clusters were identified by their corresponding marker genes.

**Figure 3.**
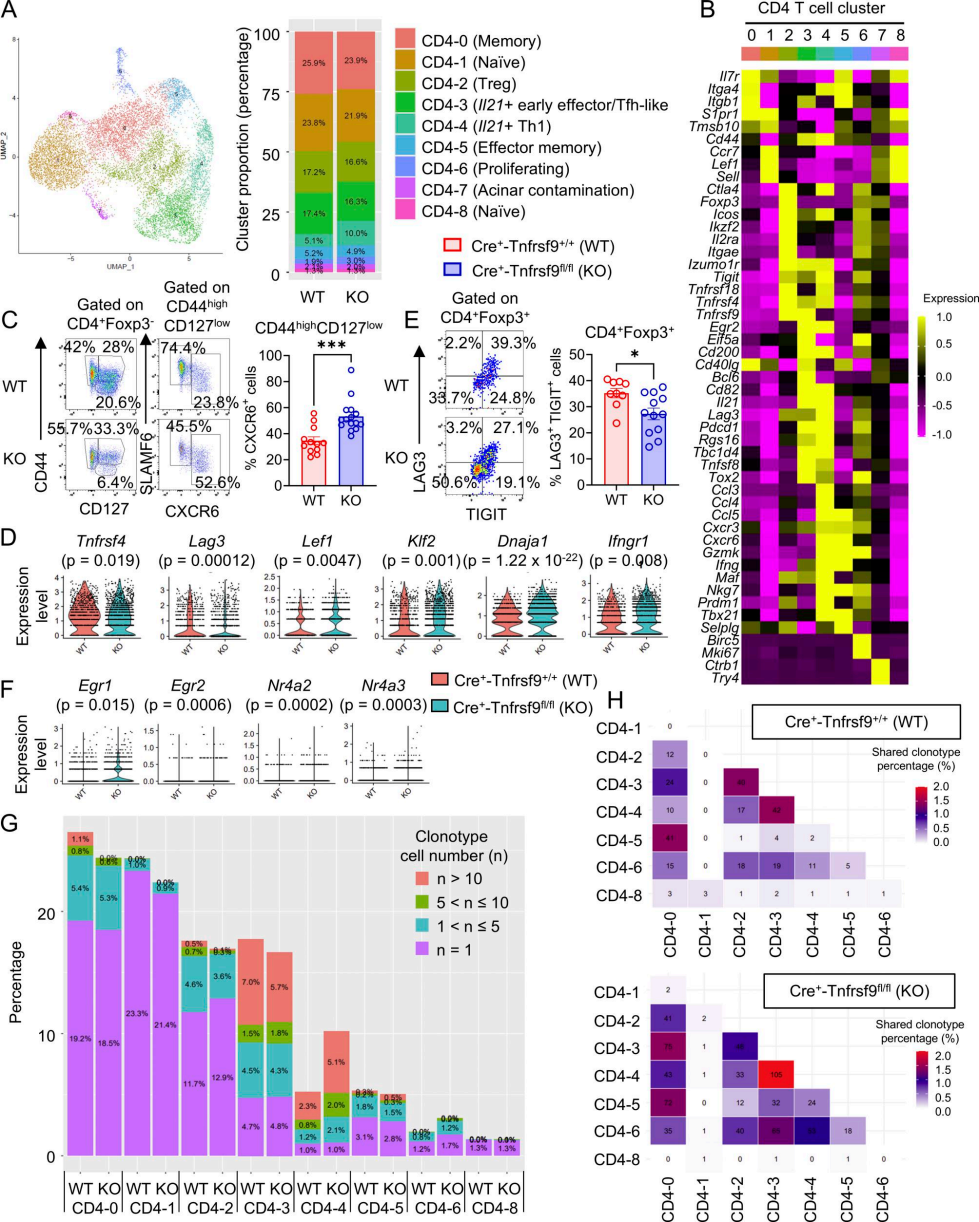
**scRNA-seq analysis reveals altered differentiation and clonal expansion of islet-infiltrating CD4 T cells in *Cre*** ^**+**^ **-*Tnfrsf9*** ^**fl/fl**^ **mice. (A)** UMAP plot of islet-infiltrating CD4 T cells isolated from 7- to 10-wk-old prediabetic *Cre*^+^-*Tnfrsf9*^+/+^ (WT) and *Cre*^+^-*Tnfrsf9*^fl/fl^ (KO) females. Bar plots show the proportions of the clusters. **(B)** Heatmap of key cluster marker genes. **(C)** Frequencies of CXCR6^+^ cells among CD44^high^CD127^low^ CD4 effector T cells in islets of 8- to 11-wk-old prediabetic *Cre*^+^-*Tnfrsf9*^+/+^ (WT) and *Cre*^+^-*Tnfrsf9*^fl/fl^ (KO) females. Representative flow cytometry profiles (left) and summarized results (right) from six experiments are shown. ***P < 0.0005, unpaired *t* test. **(D)** Differential expression of *Tnfrsf4*, *Lag3*, *Lef1*, *Klf2*, *Dnaja1*, *Ifngr1* between *Cre*^+^-*Tnfrsf9*^+/+^ (WT) and *Cre*^+^-*Tnfrsf9*^fl/fl^ (KO) cluster CD4-2 (Treg) cells. Adjusted P value for each comparison is shown in the parentheses. **(E)** Frequencies of LAG3^+^TIGIT^+^ cells within Foxp3^+^ Tregs in islets of 8- to 11-wk-old prediabetic *Cre*^+^-*Tnfrsf9*^+/+^ (WT) and *Cre*^+^-*Tnfrsf9*^fl/fl^ (KO) females. Representative flow cytometry profiles (left) and summarized results (right) from five experiments are shown. *P < 0.05 by an unpaired *t* test. **(F)** Differential expression of *Egr1*, *Egr2*, *Nr4a2*, and *Nr4a3* between *Cre*^+^-*Tnfrsf9*^+/+^ (WT) and *Cre*^+^-*Tnfrsf9*^fl/fl^ (KO) cluster CD4-1 (naïve) cells. Adjusted P value for each comparison is shown in the parentheses. **(G)** Clonal expansion of *Cre*^+^-*Tnfrsf9*^+/+^ (WT) and *Cre*^+^-*Tnfrsf9*^fl/fl^ (KO) cells within each CD4 T cell cluster. The percentages of cells in clonotypes with 1, 2–5, 6–10, or >10 cells in the indicated cluster are shown. **(H)** Tile plots showing the percentage of clonotype overlap across clusters in *Cre*^+^-*Tnfrsf9*^+/+^ (WT) and *Cre*^+^-*Tnfrsf9*^fl/fl^ (KO) CD4 T cells. The number in each tile indicates the number of unique clonotypes shared by the two clusters. The overall clonotype overlap across different clusters was increased in *Cre*^+^-*Tnfrsf9*^fl/fl^ mice (P = 0.02 compared with *Cre*^+^-*Tnfrsf9*^+/+^, Wilcoxon signed rank test).

To further determine how CD137 deficiency affects the function of islet Foxp3^+^ Tregs and the impact on conventional T cells, we performed paired single-cell RNA sequencing (scRNA-seq) and scTCR-seq. CD45.1^+^ islet-infiltrating CD4 T cells and non-CD4 T cells were sorted from 7 to 10-wk-old prediabetic *Cre*^+^-*Tnfrsf9*^+/+^ and *Cre*^+^-*Tnfrsf9*^fl/fl^ females and used for preparation of sequencing libraries (Fig. S3). After quality control filtering, we obtained 4,252 *Cre*^+^-*Tnfrsf9*^+/+^ and 7,989 *Cre*^+^-*Tnfrsf9*^fl/fl^ CD4 T cells that had both TCRα and TCRβ chain sequences. Subsequently, these CD4 T cells were grouped into nine clusters (Fig. 3 A). Identities of these cell clusters were determined by key marker genes (Fig. 3 B and Table S1) and guided by our previous scRNA-seq analysis of islet-infiltrating CD4 T cells (Ciecko et al., 2023). Cluster CD4-0 (memory) expressed *Il7r*, *S1pr1*, and *Cd44*. Clusters CD4-1/CD4-8 (naïve) expressed *Ccr7*, *Lef1*, and *Sell,* but not *Cd44*. Cluster CD4-2 (Treg) expressed genes typically found in Foxp3^+^ Tregs, including *Foxp3*, *Il2ra*, *Ikzf2*, *Tigit*, *Tnfrsf18*, and *Ctla4*. Both CD4-3 (*Il21*^+^ early effector/Tfh-like) and CD4-4 (*Il21*^+^ Th1) expressed high levels of *Il21*. While cluster CD4-3 (*Il21*^+^ early effector/Tfh-like) expressed genes associated with recent activation (*Egr2*, *Eif5a*, and *Tnfrsf9*) and Tfh cells (*Bcl6*, *Cd82*, *Tox2*, *Pdcd1*, and *Tbc1d4*), cluster CD4-4 (*Il21*^+^ Th1) expressed genes representing a Th1 phenotype (*Ccl3*, *Ccl4*, *Ccl5*, *Cxcr3*, *Cxcr6*, *Ifng*, *Nkg7*, and *Tbx21*). Cluster CD4-5 (effector memory) expressed both effector and memory markers. Genes associated with cell proliferation (*Birc5* and *Mki67*) are enriched in cluster CD4-6 (proliferating). Cluster CD4-7 was contaminated with abundant acinar cell transcripts (*Ctrb1* and *Try4*) and was excluded from subsequent analyses. Noticeably, cluster CD4-4 (*Il21*^+^ Th1) was proportionally increased from 5.1% in the *Cre*^+^-*Tnfrsf9*^+/+^ control to 10% in *Cre*^+^-*Tnfrsf9*^fl/fl^ mice, indicating that CD137 expression in Foxp3^+^ Tregs controls accumulation of this subset (Fig. 3 A). Analysis of CXCR6 expression in Foxp3^−^CD44^high^CD127^low^ CD4 effector T cells confirmed the bias toward the Th1 effector differentiation in the islets of *Cre*^+^-*Tnfrsf9*^fl/fl^ mice (Fig. 3 C).

**Figure S4.**
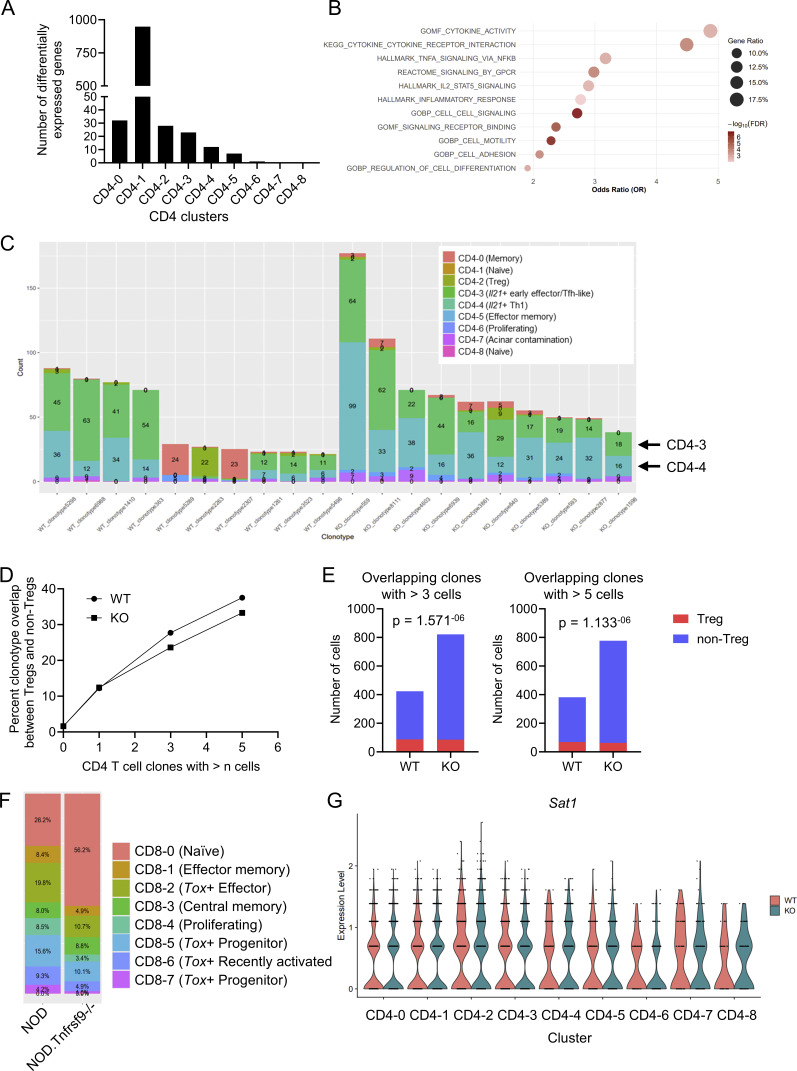
**Differential gene expression and clonotype analysis of islet-infiltrating T cells, related to Fig. 3 and 4. (A)** Number of differentially expressed genes between Cre^+^-*Tnfrsf9*^+/+^ or Cre^+^-*Tnfrsf9*^fl/fl^ mice in each islet CD4 T cell cluster. **(B)** Pathway analysis of genes upregulated in Cre^+^-*Tnfrsf9*^fl/fl^ versus Cre^+^-*Tnfrsf9*^+/+^ cluster CD4-1 (naïve) cells. **(C)** Cluster distribution of the top 10 expanded islet CD4 T cell clones in Cre^+^-*Tnfrsf9*^+/+^ (WT) and Cre^+^-*Tnfrsf9*^fl/fl^ (KO) mice. **(D)** Percentage of clonotype overlap between Foxp3^+^ Tregs and non-Tregs in Cre^+^-*Tnfrsf9*^+/+^ (WT) and Cre^+^-*Tnfrsf9*^fl/fl^ (KO) mice. The analysis was done for all clones or restricted to clones with more than one, three, or five cells. **(E)** Cell distribution in Treg or non-Treg clusters for cells within overlapping clones with more than three cells (left) or with more than five cells (right). Statistical significance was determined by the chi-square test. **(F)** Bar plots show the proportions of islet-infiltrating NOD and NOD.*Tnfrsf9*^−/−^ CD8 T cells projected onto the clusters defined in Fig. 4 A. **(G)** Expression levels of *Sat1* in Cre^+^-*Tnfrsf9*^+/+^ (WT) and Cre^+^-*Tnfrsf9*^fl/fl^ (KO) CD4 T cell clusters shown in Fig. 3 A.

*Cre* ^+^-*Tnfrsf9*^+/+^ and *Cre*^+^-*Tnfrsf9*^fl/fl^ CD4 T cells within each cluster were directly compared to identify differentially expressed genes (Table S2). In cluster CD4-2 (Treg), *Tnfrsf4* (OX40) and *Lag3* were upregulated in *Cre*^+^-*Tnfrsf9*^+/+^ cells; conversely, *Lef1*, *Klf2*, *Dnaja1*, and *Ifngr1* were upregulated in *Cre*^+^-*Tnfrsf9*^fl/fl^ cells (Fig. 3 D). *Lef1* and *Klf2* are associated with naïve and memory T cells (Sebzda et al., 2008; Willinger et al., 2006). *Dnaja1* expression and IFNγ signaling have been linked to functionally impaired Tregs (Osman et al., 2021; Overacre-Delgoffe et al., 2017). These results suggest that the differentiation status of *Cre*^+^-*Tnfrsf9*^+/+^ and *Cre*^+^-*Tnfrsf9*^fl/fl^ Tregs was different. A reduced frequency of *Cre*^+^-*Tnfrsf9*^fl/fl^ islet Tregs expressing both TIGIT and LAG3 (typically associated with their activation) indicated their less activated phenotype (Fig. 3 E). Unexpectedly, among all clusters, CD4-1 (naïve) cells had the highest number of differentially expressed genes between *Cre*^+^-*Tnfrsf9*^+/+^ and *Cre*^+^-*Tnfrsf9*^fl/fl^ (Fig. S4 A). Although the overall expression levels of *Egr1*, *Egr2*, *Nr4a2*, and *Nr4a3* were low in CD4-1 (naïve) cluster cells, they were among the genes upregulated in *Cre*^+^-*Tnfrsf9*^fl/fl^ cells compared with the *Cre*^+^-*Tnfrsf9*^+/+^ counterparts (Fig. 3 F), suggesting elevated TCR signaling in a subset of the former population. Pathway analysis revealed that genes upregulated in *Cre*^+^-*Tnfrsf9*^fl/fl^ CD4-1 (naïve) cluster cells were enriched in cytokine and cellular activities (Fig. S4 B), supporting that they were more positioned to become effector cells than the counterparts in *Cre*^+^-*Tnfrsf9*^+/+^ mice.

Next, we used the TCR sequence as a barcode and asked whether Foxp3^+^ Treg–specific knockout of CD137 alters CD4 T cell clonal expansion and differentiation. A clonotype is defined by the paired TCRα and TCRβ V/J genes and CDR3 nucleotide sequences. As expected, cells from expanded clones were primarily present in activated cell clusters (Fig. 3 G). Of note, a higher frequency of cells from expanded clones was found in cluster CD4-4 (*Il21*^+^ Th1) of *Cre*^+^-*Tnfrsf9*^fl/fl^ than *Cre*^+^-*Tnfrsf9*^+/+^ mice. As T cells bearing the same TCRs in two different clusters reflect their differentiation from one state to another or from the same early precursors, we evaluated CD4 T cell differentiation potential by clonotype overlap across cell clusters. This analysis revealed that the overall clonotype overlap across different clusters was increased in *Cre*^+^-*Tnfrsf9*^fl/fl^ mice (P = 0.02, Wilcoxon’s signed rank test, Fig. 3 H). One exception was between CD4-2 (Treg) and CD4-3 (*Il21*^+^ early effector/Tfh-like) clusters. When considering only clonotypes within clusters CD4-2 and CD4-3, the level of overlap was higher in *Cre*^+^-*Tnfrsf9*^+/+^ than in *Cre*^+^-*Tnfrsf9*^fl/fl^ mice (P = 0.016, chi-square test). Analysis of the top 10 expanded CD4 T cell clones in each strain revealed that most of the cells were in clusters CD4-3 (*Il21*^+^ early effector/Tfh-like) and CD4-4 (*Il21*^+^ Th1) except for three clones (clonotypes 5289, 2263, and 2307 from *Cre*^+^-*Tnfrsf9*^+/+^ mice) that had most cells in cluster CD4-2 (Treg) or CD4-0 (memory) (Fig. S4 C). Next, we asked whether the clonotype overlap between CD4-2 (Treg) and all non-Treg clusters is different between *Cre*^+^-*Tnfrsf9*^+/+^ and *Cre*^+^-*Tnfrsf9*^fl/fl^ mice. Clusters CD4-6 (proliferating) and CD4-7 (acinar contamination) were excluded from this analysis as they may contain Tregs. This analysis revealed a high frequency (>10%) of clonotype overlap between Tregs and non-Tregs in expanded clones (more than one cell) in both strains (Fig. S4 D). While not statistically significant, a trend of higher clonotype overlap was observed in *Cre*^+^-*Tnfrsf9*^+/+^ than in *Cre*^+^-*Tnfrsf9*^fl/fl^ mice in clones with more than three cells (Fig. S4 D). Interestingly, cells of the clonotypes present in both Tregs and non-Tregs were more likely to be non-Tregs in *Cre*^+^-*Tnfrsf9*^fl/fl^ than in *Cre*^+^-*Tnfrsf9*^+/+^ mice (Fig. S4 E). Collectively, these results indicate that CD4 T cells in *Cre*^+^-*Tnfrsf9*^fl/fl^ mice more actively differentiated into effector cells with a Th1 phenotype.

**Figure 4.**
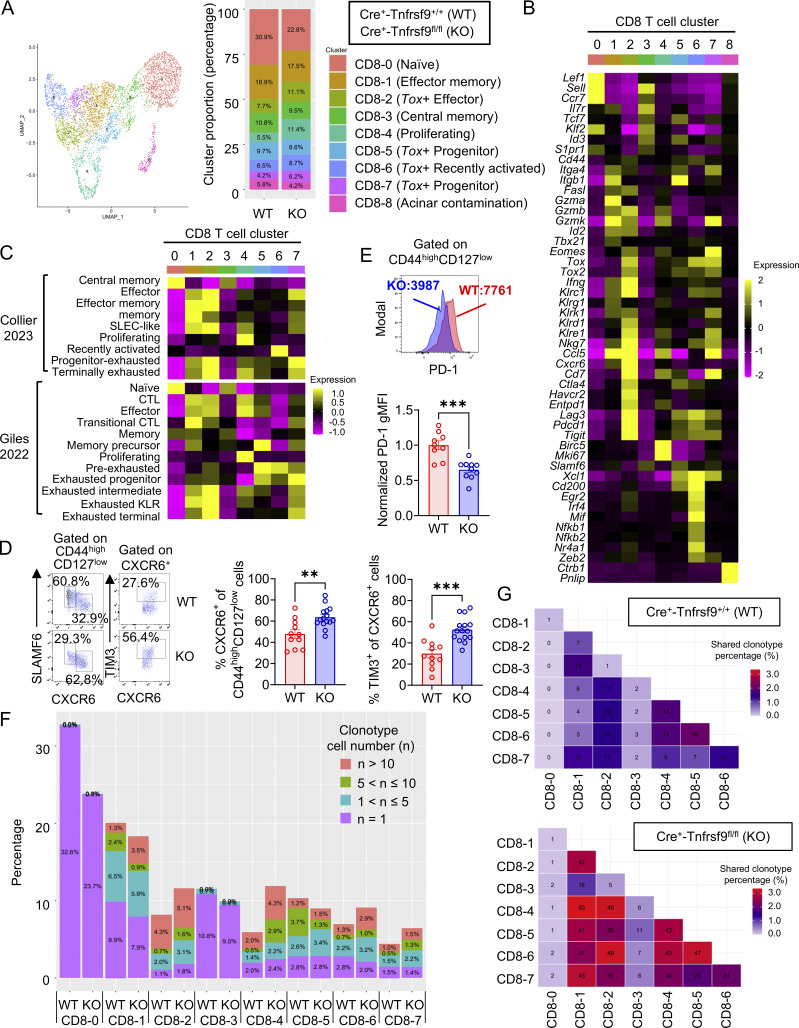
**scRNA-seq analysis reveals altered differentiation and clonal expansion of islet-infiltrating CD8 T cells in *Cre*** ^**+**^ **-*Tnfrsf9*** ^***f*l/fl**^ **mice. (A)** UMAP plot of islet-infiltrating CD8 T cells isolated from 7- to 10-wk-old prediabetic *Cre*^+^-*Tnfrsf9*^+/+^ (WT) and *Cre*^+^-*Tnfrsf9*^fl/fl^ (KO) females. Bar plots show the proportions of the clusters. **(B)** Heatmap of key cluster marker genes. **(C)** Gene module scores of the signatures obtained from previously defined CD8 T cell differentiation states in T1D (Collier et al., 2023) and viral infection (Giles et al., 2022). **(D)** Frequencies of CXCR6^+^ cells among CD44^high^CD127^low^ and TIM3^+^ cells among CD44^high^CD127^low^CXCR6^+^ CD8 effector T cells in islets of 8- to 11-wk-old prediabetic *Cre*^+^-*Tnfrsf9*^+/+^ (WT) and *Cre*^+^-*Tnfrsf9*^fl/fl^ (KO) females. Representative flow cytometry profiles (left) and summarized results (right) from six experiments are shown. **P < 0.005; ***P < 0.0005, unpaired *t* test. **(E)** Relative PD-1 gMFI of CD44^high^CD127^low^ CD8 T cells from the islets of 8- to 11-wk-old prediabetic *Cre*^+^-*Tnfrsf9*^+/+^ (WT) and *Cre*^+^-*Tnfrsf9*^fl/fl^ (KO) females. Representative flow cytometry profiles (upper) and summarized results (lower) from four experiments are shown. ***P < 0.0005, unpaired *t* test. **(F)** Clonal expansion of *Cre*^+^-*Tnfrsf9*^+/+^ (WT) and *Cre*^+^-*Tnfrsf9*^fl/fl^ (KO) cells within each CD8 T cell cluster. The percentages of cells in clonotypes with 1, 2–5, 6–10, or >10 cells in the indicated cluster are shown. **(G)** Tile plots showing the percentage of clonotype overlap across clusters in *Cre*^+^-*Tnfrsf9*^+/+^ (WT) and *Cre*^+^-*Tnfrsf9*^fl/fl^ (KO) CD8 T cells. The number in each tile indicates the number of unique clonotypes shared by the two clusters. Clonotype overlap across cell clusters was higher in *Cre*^+^-*Tnfrsf9*^fl/fl^ than in *Cre*^+^-*Tnfrsf9*^*+/+*^ mice (P = 1.759 × 10^−05^, Wilcoxon signed rank test). gMFI, geometric mean fluorescence intensity.

Within islet non-CD4 T cells, CD8 T cell clusters, identified by *Cd3d*, *Cd8a*, and *Cd8b1* expression, were found to trend proportionally higher in *Cre*^+^-*Tnfrsf9*^fl/fl^ than in *Cre*^+^-*Tnfrsf9*^+/+^ mice (Fig. S3). We obtained 1,301 *Cre*^+^-*Tnfrsf9*^+/+^ and 3,159 *Cre*^+^-*Tnfrsf9*^fl/fl^ CD8 T cells that had both TCRα and TCRβ chain sequences and separated them into nine clusters (Fig. 4 A). The differentiation states of these clusters were defined based on key marker genes and module scores of previously reported CD8 T cell gene signatures (Fig. 4, B and C; and Table S3) (Collier et al., 2023; Giles et al., 2022). Cluster CD8-0 (naïve) expressed *Lef1*, *Sell*, and *Ccr7*. CD8-1 (effector memory) cluster cells expressed *Il7r*, *Itgb1*, *Ifng*, *Gzma*, *Gzmk*, and *Ccl5*, and had high module scores for both effector and memory gene signatures. Cluster CD8-3 (central memory) cells expressed *Sell*, *Il7r*, and *Klf2*, and had high gene module scores for naïve and memory CD8 T cells. The expression of *Birc5* and *Mki67* defined cluster CD8-4 (proliferating). *Tox* was expressed in clusters CD8-2 (*Tox*^+^ effector), CD8-5 (*Tox*^+^ progenitor), CD8-6 (*Tox*^+^ recently activated), and CD8-7 (*Tox*^+^ progenitor), suggesting that they were under chronic antigen stimulation. Cluster CD8-2 (*Tox*^+^ effector) cells expressed high levels of *Gzmb*, *Ifng*, *Klrc1*, *Klre1*, *Nkg7*, *Ccl5*, and *Cxcr6*, as well as co-inhibitory molecules *Ctla4*, *Havcr2* (TIM3), *Entpd1* (CD39), *Lag3*, and *Pdcd1* (PD-1), but lacked the expression of *Tcf7* (TCF1) and *Slamf6*, resembling both CX3CR1^+^ effector and terminally exhausted CD8 T cells found during chronic viral infection (Zander et al., 2019). Compared with cluster CD8-2 (*Tox*^+^ effector), both CD8-5 (*Tox*^+^ progenitor) and CD8-7 (*Tox*^+^ progenitor) expressed lower levels of co-inhibitory molecules but higher *Tcf7* and *Slamf6*. Gene module score analysis suggested that cluster CD8-5 (*Tox*^+^ progenitor) cells are close to memory precursors and pre-exhausted CD8 T cells respectively found at the early stage of acute and chronic viral infection, suggesting that cells within CD8-5 (*Tox*^+^ progenitor) and CD8-7 (*Tox*^+^ progenitor) clusters were at different stages of differentiation (Giles et al., 2022). Cluster CD8-6 (*Tox*^+^ recently activated) cells expressed *Egr2*, *Irf4*, *Nfkb1*, *Nfkb2*, and *Nr4a1* associated with TCR signaling and had a high module score for the recently activated signature (Collier et al., 2023). Cluster CD8-8 was contaminated with abundant acinar cell transcripts (*Ctrb1* and *Pnlip*) and was subsequently excluded from analysis.

Relative to the wild type, CD8 T cells from *Cre*^+^-*Tnfrsf9*^fl/fl^ mice were proportionally reduced in CD8-0 (naïve) and increased in CD8-4 (proliferating) clusters (Fig. 4 A), consistent with the flow cytometry results (Fig. 2, C and E). Of note, the fate of *Cre*^+^-*Tnfrsf9*^fl/fl^ CD8 T cells was skewed toward *Tox*^+^ clusters, especially the more terminally differentiated CD8-2 (*Tox*^+^ effector) cells (Fig. 4 A). This bias was confirmed by flow cytometry analysis showing elevated percentage of CXCR6^+^ cells within CD44^high^CD127^low^ effectors and the frequency of TIM3^+^ cells among the CXCR6^+^ population in islets of *Cre*^+^-*Tnfrsf9*^fl/fl^ mice (Fig. 4 D). Despite having a higher percentage of CXCR6^+^TIM3^+^ terminally differentiated effectors within islet CD44^high^CD127^low^ CD8 T cells in *Cre*^+^-*Tnfrsf9*^fl/fl^ mice, their PD-1 expression was significantly reduced when compared to the wild-type counterpart (Fig. 4 E), suggesting that they were less impacted by PD-1–mediated suppression. We previously observed that CD137 expression by CD8 T cells was important for their proliferation and diabetogenic activity (Forsberg et al., 2017). To further study the role of CD137 in CD8 T cells, we isolated them from the islets of 9- to 10-wk-old prediabetic *Tnfrsf9*^−/−^ global knockout females and the corresponding wild-type control and compared them by scRNA-seq analysis. CD8 T cells from this latter scRNA-seq experiment were projected onto the Uniform Manifold Approximation and Projection (UMAP) in Fig. 4 A to maintain the same cluster identity. Interestingly, CD8 T cells lacking CD137 preferentially accumulated in the naïve cluster and had a reduced proportion of the proliferating cluster, and their fate was skewed away from the *Tox*^+^ terminally differentiated state (Fig. S4 F). Thus, CD137 signaling in CD8 T cells promoted their proliferation and terminal differentiation, and these effects were further exaggerated in *Cre*^+^-*Tnfrsf9*^fl/fl^ mice where CD137 is only deleted in Foxp3^+^ Tregs. Of note, T1D development in CD137 global knockout NOD mice is delayed (Chen et al., 2014). Thus, these collective results also indicate that in the absence of a strong diabetogenic CD8 T cell response, CD137-dependent Foxp3^+^ Treg–mediated suppression becomes less important.

TCR sequence analysis revealed that cells from the expanded clones were mostly found in activated clusters, including those expressing *Tox* (Fig. 4 F). A tendency of increased frequencies of cells from the expanded clones, especially in the CD8-4 (proliferating) cluster, was observed in *Cre*^+^-*Tnfrsf9*^fl/fl^ mice. Clonotype overlap across cell clusters was higher in *Cre*^+^-*Tnfrsf9*^fl/fl^ than in *Cre*^+^-*Tnfrsf9*^*+/+*^ mice (P = 1.759 × 10^−05^, Wilcoxon’s signed rank test, Fig. 4 G). Interestingly, there was appreciable overlap between *Tox*^−^ CD8-1 (effector memory) and all *Tox*^+^ clusters in *Cre*^+^-*Tnfrsf9*^fl/fl^ mice, supporting an elevated recruitment of autoreactive CD8 T cells into inflamed islets. Together, the results suggest that islet-infiltrating CD8 T cells in *Cre*^+^-*Tnfrsf9*^fl/fl^ mice were more proliferative and actively differentiated from early to terminal effectors than their counterparts in the wild-type control.

### CD137 deficiency alters Treg clonal expansion and differentiation trajectory

**Figure 5.**
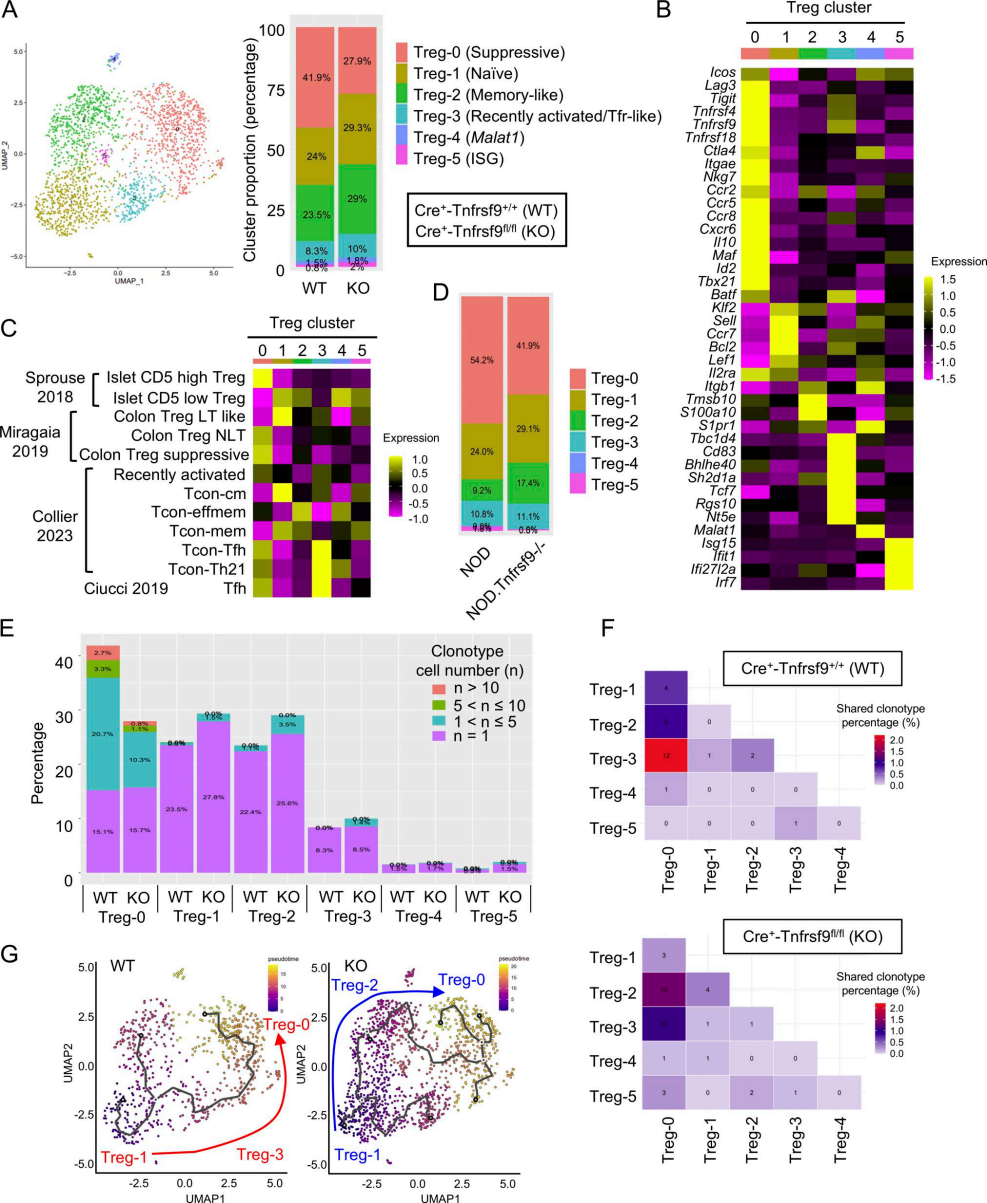
**scRNA-seq analysis reveals altered differentiation and clonal expansion of islet-infiltrating Foxp3** ^**+**^ **Tregs in *Cre*** ^**+**^ **-*Tnfrsf9*** ^**fl/fl**^ **mice. (A)** UMAP plot of islet-infiltrating Foxp3^+^ T cells reclustered from CD4-2 (Treg) cells shown in Fig. 3 A. Bar plots show the proportions of the clusters. **(B)** Heatmap of key cluster marker genes. **(C)** Gene module scores of the signatures obtained from the previously defined Foxp3^+^ Treg (Sprouse et al., 2018; Miragaia et al., 2019) and conventional CD4 T cell (Collier et al., 2023) differentiation states. **(D)** Bar plots show the proportions of islet-infiltrating WT NOD and NOD.*Tnfrsf9*^−/−^ Foxp3^+^ Tregs projected onto the clusters defined in A. **(E)** Clonal expansion of *Cre*^+^-*Tnfrsf9*^+/+^ (WT) and *Cre*^+^-*Tnfrsf9*^fl/fl^ (KO) cells within each Treg cluster. The percentages of cells in clonotypes with 1, 2–5, 6–10, or >10 cells in the indicated cluster are shown. **(F)** Tile plots showing the percentage of clonotype overlap across clusters in *Cre*^+^-*Tnfrsf9*^+/+^ (WT) and *Cre*^+^-*Tnfrsf9*^fl/fl^ (KO) Tregs. The number in each tile indicates the number of unique clonotypes shared by the two clusters. **(G)** Differentiation trajectory of *Cre*^+^-*Tnfrsf9*^+/+^ (WT) and *Cre*^+^-*Tnfrsf9*^fl/fl^ (KO) Tregs.

**Figure S5.**
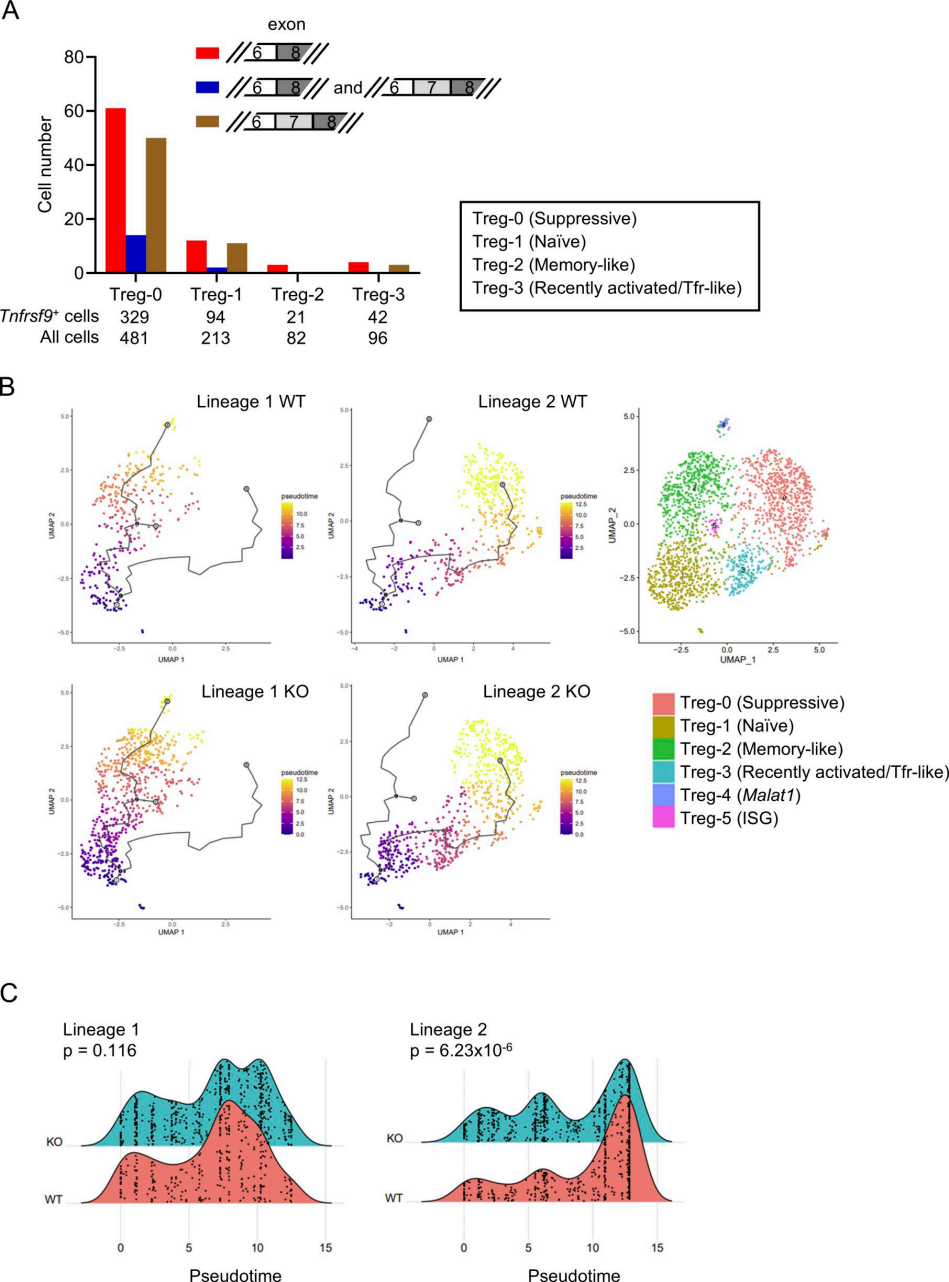
**Analysis of islet Foxp3** ^**+**^ **Treg cluster cells, related to Fig. 5. (A)** Analysis of *Tnfrsf9* exon 7 (transmembrane domain–encoding exon) alternative splicing in Foxp3^+^ Tregs of different differentiation states using WT cells shown in Fig. 5 D. The bar plot only contains cells with informative reads mapped to *Tnfrsf9* exons 6/7, 7/8, or 6/8. **(B)** Cre^+^-*Tnfrsf9*^+/+^ (WT) and Cre^+^-*Tnfrsf9*^fl/fl^ (KO) Foxp3^+^ Tregs were combined and analyzed for differentiation pseudotime by Monocle 3. UMAPs of lineages 1 and 2 of Cre^+^-*Tnfrsf9*^+/+^ (WT) and Cre^+^-*Tnfrsf9*^fl/fl^ (KO) Foxp3^+^ Treg clusters are shown. **(C)** Distribution of WT or KO Foxp3^+^ Tregs along the pseudotime of lineage 1 or 2. Statistical significance was determined by the Kolmogorov–Smirnov test.

To gain further insights into the differentiation states of Foxp3^+^ Tregs, we reclustered the CD4-2 (Treg) cluster (733 *Cre*^+^-*Tnfrsf9*^+/+^ and 1,326 *Cre*^+^-*Tnfrsf9*^fl/fl^ cells) into six subpopulations (Fig. 5, A and B; and Table S4). Cluster Treg-0 upregulated *Icos*, *Lag3*, *Tigit*, *Ctla4*, and *Itgae*, typically associated with highly suppressive Tregs. Cluster Treg-1 expressed high levels of *Klf2*, *Sell*, *Ccr7*, and *Lef1*, indicating a naïve phenotype. The differentiation states of clusters Treg-2 and Treg-3 were less definitive, with high levels of *Klf2*, *Itgb1*, *S100a10*, and *S1pr1* in the former and *Tbc1d4*, *Cd83*, *Rgs10*, *Bhlhe40*, *Tcf7*, and *Nt5e* in the latter populations. The two minor clusters Treg-4 and Treg-5 respectively express high levels of *Malat1* and IFN-stimulated genes. The differentiation states of these Treg clusters were further examined by module score analysis using gene sets associated with distinct Foxp3^+^ Treg and CD4 T cell subpopulations (Fig. 5 C) (Collier et al., 2023; Miragaia et al., 2019; Sprouse et al., 2018). Cluster Treg-0 cells were found most similar to islet CD5^high^ Tregs that had a potent T1D suppressing activity (Sprouse et al., 2018) and the highly differentiated tissue Tregs found in colon (Miragaia et al., 2019). Cluster Treg-1 cells were enriched for genes expressed in islet CD5^low^ Tregs, colon lymphoid tissue-like Tregs, and central memory T cells, consistent with them being naïve Tregs. Cells within cluster Treg-2 were similar to islet CD5^low^ Tregs and effector memory T cells, suggesting a memory-like phenotype. Cluster Treg-3 cells were more closely related to recently activated and Tfh cells, suggesting that they contained Tregs in an early stage of activation, as well as Tfr-like cells. Treg-4 and Treg-5 cells appeared to be mixed populations of less differentiated Tregs. Compared with the *Cre*^+^-*Tnfrsf9*^+/+^ control, *Cre*^+^-*Tnfrsf9*^fl/fl^ mice were proportionally reduced in cluster Treg-0, suggesting that CD137 expressed in Tregs controlled their differentiation. Nevertheless, increased autoimmune inflammation in islets of *Cre*^+^-*Tnfrsf9*^fl/fl^ mice may have a negative impact on Treg differentiation. We previously showed that global CD137-deficient (*Tnfrsf9*^−/−^) NOD mice had similar insulitis at 10 wk of age but delayed T1D onset when compared to the wild type (Chen et al., 2014). Thus, we compared Foxp3^+^ Tregs from the islets of 9- to 10-wk-old prediabetic *Tnfrsf9*^−/−^ females and the corresponding wild-type control by scRNA-seq analysis. Tregs from this latter scRNA-seq experiment were projected onto the UMAP in Fig. 5 A. Similar to Tregs from *Cre*^+^-*Tnfrsf9*^fl/fl^ mice, those from the *Tnfrsf9*^−/−^ strain were also less frequently present in cluster Treg-0 than the corresponding wild-type counterparts (Fig. 5 D), confirming a role of CD137 in Treg differentiation. Next, we developed an R package, exonBlocks, to determine whether the differentiation states of Foxp3^+^ Tregs influenced the alternative splicing of exon 7 (encoding the transmembrane domain). Exon 7 alternative splicing was found to occur in all cell clusters without an apparent preference (Fig. S5 A). It was noted that many cells preferentially expressed either the soluble or the membrane form (i.e., excluding or including exon 7, respectively). However, this observation is likely due to a low number of *Tnfrsf9* unique molecular identifiers (UMIs) that were recovered from *Tnfrsf9*^+^ cells and contained informative reads.

Next, *Cre*^+^-*Tnfrsf9*^+/+^ and *Cre*^+^-*Tnfrsf9*^fl/fl^ Tregs were further compared by TCR clonotype analysis. Most cells of expanded clones were in cluster Treg-0 (suppressive), consistent with their highly activated phenotype (Fig. 5 E). Interestingly, within the Treg-0 (suppressive) cluster, a higher frequency of cells from expanded clones was found in *Cre*^+^-*Tnfrsf9*^+/+^ mice compared with the counterpart of *Cre*^+^-*Tnfrsf9*^fl/fl^ mice. Conversely, the frequency of cells from expanded clones in Treg-1, Treg-2, and Treg-3 clusters trended higher in *Cre*^+^-*Tnfrsf9*^fl/fl^ mice. Clonotype overlap among different Treg clusters was analyzed to determine developmental relatedness (Fig. 5 F). Of note, the highest clonotype overlap was found between clusters Treg-0 (suppressive) and Treg-3 (recently activated/Tfr-like) in *Cre*^+^-*Tnfrsf9*^+/+^ Tregs, but it was between Treg-0 (suppressive) and Treg-2 (memory-like) in the *Cre*^+^-*Tnfrsf9*^fl/fl^ counterparts (Fig. 5 F), suggesting an alteration of their differentiation pathways in the absence of CD137. To further test this possibility, we determined the differentiation trajectory of *Cre*^+^-*Tnfrsf9*^+/+^ and *Cre*^+^-*Tnfrsf9*^fl/fl^ Tregs separately using Monocle 3 (Cao et al., 2019). This analysis revealed that the main differentiation path for *Cre*^+^-*Tnfrsf9*^+/+^ Tregs was from Treg-1 (naïve) to Treg-3 (recently activated/Tfr-like) and then to Treg-0 (suppressive). However, the main trajectory of *Cre*^+^-*Tnfrsf9*^fl/fl^ Treg differentiation was from Treg-1 (naïve) to Treg-2 (memory-like) and then to Treg-0 (suppressive) (Fig. 5 G). Having determined the individual differentiation trajectory, we analyzed both *Cre*^+^-*Tnfrsf9*^+/+^ and *Cre*^+^-*Tnfrsf9*^fl/fl^ Tregs together to directly compare their pseudotemporal progression. Differentiation lineages 1 and 2 were identified that respectively passed through clusters Treg-2 (memory-like) and Treg-3 (recently activated/Tfr-like) (Fig. S5 B). Distribution of *Cre*^+^-*Tnfrsf9*^+/+^ and *Cre*^+^-*Tnfrsf9*^fl/fl^ Tregs along lineage 1 pseudotime was similar (Fig. S5 C). However, more *Cre*^+^-*Tnfrsf9*^fl/fl^ Tregs were located within the first half, and conversely, more *Cre*^+^-*Tnfrsf9*^+/+^ cells were in the second half of lineage 2 pseudotime, indicating a paucity of CD137-deficient Foxp3^+^ Tregs to become fully activated (Fig. S5 C).

### Soluble CD137 produced by Foxp3^+^ Tregs suppresses T1D development

**Figure 6.**
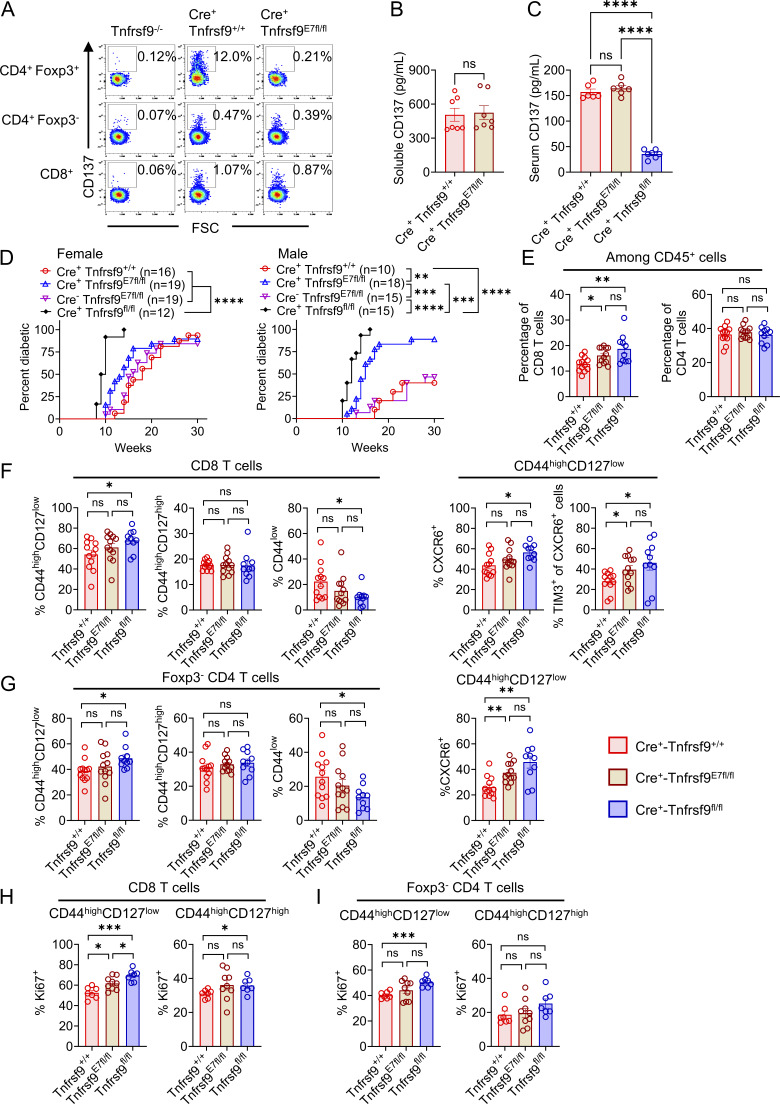
**Foxp3** ^**+**^ **Tregs mediate T1D suppression through soluble CD137. (A)** CD137 expression in splenic Foxp3^+^ and Foxp3^−^ CD4 T cells and CD8 T cells of *Cre*^+^-*Tnfrsf9*^+/+^ and *Cre*^+^-*Tnfrsf9*^E7fl/fl^ mice. NOD.*Tnfrsf9*^−/−^ cells were used as the negative control. Representative flow cytometry profiles of three mice per genotype are shown. **(B)** Soluble CD137 produced by cultured *Cre*^+^-*Tnfrsf9*^+/+^ and *Cre*^+^-*Tnfrsf9*^E7fl/fl^ splenic Foxp3^+^ Tregs (*n* = 7). ns: not significant. **(C)** Circulating soluble CD137 in 7- to 9-wk-old *Cre*^+^-*Tnfrsf9*^+/+^, *Cre*^+^-*Tnfrsf9*^E7fl/fl^, and *Cre*^+^-*Tnfrsf9*^fl/fl^ males. ****P < 0.0001 by an unpaired *t* test. **(D)** T1D incidence study of *Cre*^+^-*Tnfrsf9*^fl/fl^ mice and *Cre*^+^-*Tnfrsf9*^+/+^, *Cre*^+^-*Tnfrsf9*^E7fl/fl^, and *Cre*^−^-*Tnfrsf9*^E7fl/fl^ female (left) and male (right) littermates. *P < 0.05; **P < 0.005; ***P < 0.0005; ****P < 0.0001 by a log-rank test. **(E)** Frequencies of islet-infiltrating CD4 and CD8 T cells in 8- to 10-wk-old prediabetic *Cre*^+^-*Tnfrsf9*^+/+^, *Cre*^+^-*Tnfrsf9*^E7fl/fl^, and *Cre*^+^-*Tnfrsf9*^fl/fl^ females. **(F and G)** Frequencies of CD8 (F) and Foxp3^−^ CD4 (G) T cell subsets in islets of 8- to 10-wk-old prediabetic *Cre*^+^-*Tnfrsf9*^+/+^, *Cre*^+^-*Tnfrsf9*^E7fl/fl^, and *Cre*^+^-*Tnfrsf9*^fl/fl^ females. **(H and I)** Frequencies of Ki67^+^ cells in CD8 (H) and Foxp3^−^ CD4 (I) T cell subsets in islets of 8- to 10-wk-old prediabetic *Cre*^+^-*Tnfrsf9*^+/+^, *Cre*^+^-*Tnfrsf9*^E7fl/fl^, and *Cre*^+^-*Tnfrsf9*^fl/fl^ females. *P < 0.05; **P < 0.005; ***P < 0.0005 by an unpaired *t* test. ns: not significant. Results shown for E–I are summarized from five to seven experiments.

Having demonstrated that complete CD137 deletion in Foxp3^+^ Tregs triggered hyperactivation of islet-infiltrating CD4 and CD8 T cells and rapid T1D onset, we sought to determine whether retaining their soluble CD137 production but not the membrane form is sufficient to alleviate the disease. To test this, we generated a second *Tnfrsf9* conditional knockout allele (*Tnfrsf9*^E7fl/fl^) that allowed us to delete cell surface CD137 but retain the soluble form by removing the transmembrane domain–encoding exon 7. The NOD.*Tnfrsf9*^E7fl/fl^ strain was then crossed to the NOD.*Foxp3*-*Cre* stock to generate mice where Foxp3^+^ Tregs lacked membrane CD137 (designated *Cre*^+^-*Tnfrsf9*^E7fl/fl^). Specific deletion of cell surface CD137 was confirmed in Foxp3^+^ Tregs (Fig. 6 A). Importantly, Foxp3^+^ Tregs isolated from *Cre*^+^-*Tnfrsf9*^+/+^ and *Cre*^+^-*Tnfrsf9*^E7fl/fl^ mice produced comparable levels of soluble CD137 in culture (Fig. 6 B). Serum CD137 in *Cre*^+^-*Tnfrsf9*^E7fl/fl^ mice was also restored to a level similar to that detected in the wild-type control (Fig. 6 C). In both females and males, retaining the ability of Foxp3^+^ Tregs to produce soluble CD137 in *Cre*^+^-*Tnfrsf9*^E7fl/fl^ mice delayed T1D development when compared to *Cre*^+^-*Tnfrsf9*^fl/fl^ mice where Foxp3^+^ Tregs lacked both soluble and cell surface CD137 (Fig. 6 D). When compared to their wild-type littermate controls, T1D development was accelerated in *Cre*^+^-*Tnfrsf9*^E7fl/fl^ males (Fig. 6 D). While the overall T1D incidence did not differ between *Cre*^+^-*Tnfrsf9*^E7fl/fl^ females and their wild-type littermate controls, the average time to diabetes onset was shorter in *Cre*^+^-*Tnfrsf9*^E7fl/fl^ than *Cre*^+^-*Tnfrsf9*^+/+^ littermates (13.82 ± 0.96 versus 18.33 ± 1.2 wk; P < 0.05, unpaired *t* test).

Analysis of islet-infiltrating T cells by flow cytometry revealed less changes in *Cre*^+^-*Tnfrsf9*^E7fl/fl^ than *Cre*^+^-*Tnfrsf9*^fl/fl^ females, when compared to the *Cre*^+^-*Tnfrsf9*^+/+^ wild-type control (Fig. 6, E–I). The percentage of CD8 T cells was higher in the islets of *Cre*^+^-*Tnfrsf9*^E7fl/fl^ mice than the *Cre*^+^-*Tnfrsf9*^+/+^ control, albeit the magnitude of increase was not as significant as that observed in *Cre*^+^-*Tnfrsf9*^fl/fl^ mice (Fig. 6 E). Based on the CD44 and CD127 expression patterns, the frequencies of effector, memory, and naïve subsets of CD8 and Foxp3^−^ CD4 T cells were comparable between *Cre*^+^-*Tnfrsf9*^+/+^ and *Cre*^+^-*Tnfrsf9*^E7fl/fl^ females (Fig. 6, F and G). Similar to *Cre*^+^-*Tnfrsf9*^fl/fl^ mice, the percentages of terminally differentiated CD8 T cells (TIM3^+^ among CXCR6^+^CD44^high^CD127^low^) and Th1 CD4 T effector cells (CXCR6^+^ among CD44^high^CD127^low^) were also higher in *Cre*^+^-*Tnfrsf9*^E7fl/fl^ mice (Fig. 6, F and G). Proliferation of CD8 and Foxp3^−^ CD4 T cells was mostly similar between *Cre*^+^-*Tnfrsf9*^+/+^ and *Cre*^+^-*Tnfrsf9*^E7fl/fl^ strains (Fig. 6, H and I). While the frequency of Ki67^+^ cells among islet CD44^high^CD127^low^ CD8 T effectors was increased in *Cre*^+^-*Tnfrsf9*^E7fl/fl^ mice relative to the *Cre*^+^-*Tnfrsf9*^+/+^ control, it was reduced when compared to *Cre*^+^-*Tnfrsf9*^fl/fl^ mice. Collectively, these results provide direct *in vivo* evidence to demonstrate that soluble CD137 produced by Foxp3^+^ Tregs is immunosuppressive and membrane CD137 expressed on Foxp3^+^ Tregs also contributes to their T1D suppressive function.

### Cell surface CD137 regulates the differentiation of islet Foxp3^+^ Tregs

**Figure 7.**
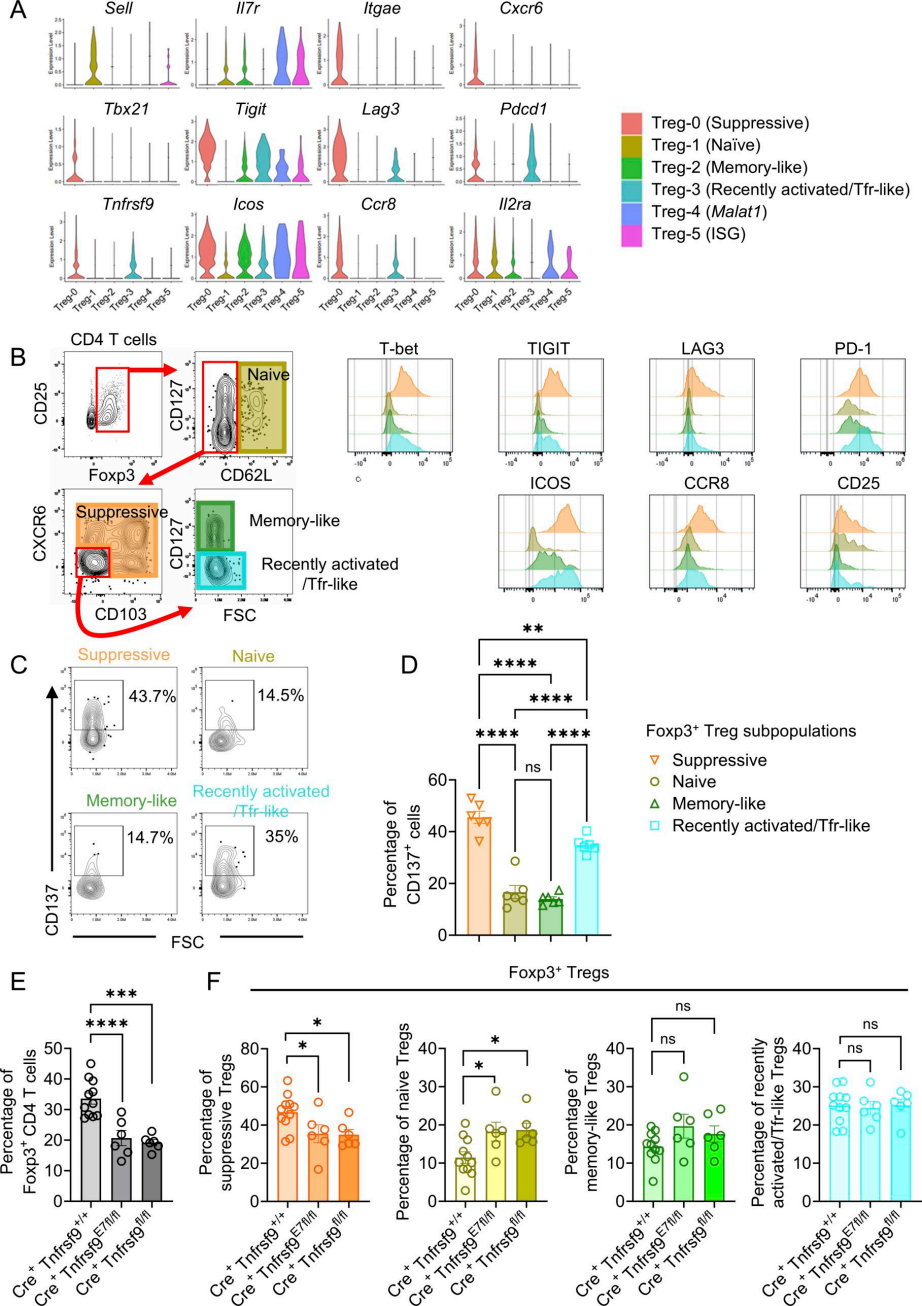
**Subset analysis of islet Foxp3** ^**+**^ **Tregs. (A)** Violin plots showing the expression levels of select markers in the suppressive, naïve, memory-like, and recently activated/Tfr-like Foxp3^+^ Treg clusters shown in Fig. 5 A. **(B)** Gating strategy defining naïve (CD62L^+^), suppressive (CD62L^−^ CD103^+^ and/or CXCR6^+^), memory-like (CD62L^−^CD103^−^CXCR6^−^CD127^+^), and recently activated/Tfr-like (CD62L^−^CD103^−^CXCR6^−^CD127^−^) subpopulations of islet Foxp3^+^ Tregs in WT NOD mice and their expression patterns of T-bet, TIGIT, LAG3, PD-1, ICOS, CCR8, and CD25. **(C and D)** CD137 expression in suppressive, naïve, memory-like, and recently activated/Tfr-like subpopulations of islet Foxp3^+^ Tregs in WT NOD mice. **(C)** Representative flow cytometry plots showing CD137 expression in the four subpopulations of islet Foxp3^+^ Tregs. **(D)** Summarized results from two experiments. **P < 0.005; ****P < 0.0001 by one-way ANOVA followed by Tukey’s multiple comparisons test. ns: not significant. **(E)** Frequencies of Foxp3^+^ Tregs among CD4 T cells in the islets of *Cre*^+^-*Tnfrsf9*^+/+^, *Cre*^+^-*Tnfrsf9*^E7fl/fl^, and *Cre*^+^-*Tnfrsf9*^fl/fl^ mice. Combined results from more than two experiments are shown. ***P < 0.0005; ****P < 0.0001 by an unpaired *t* test. **(F)** Frequencies of suppressive, naïve, memory-like, and recently activated/Tfr-like subpopulations of Foxp3^+^ Tregs in the islets of *Cre*^+^-*Tnfrsf9*^+/+^, *Cre*^+^-*Tnfrsf9*^E7fl/fl^, and *Cre*^+^-*Tnfrsf9*^fl/fl^ mice. Combined results from more than two experiments are shown. *P < 0.05 by an unpaired *t* test. ns: not significant.

**Figure 8.**
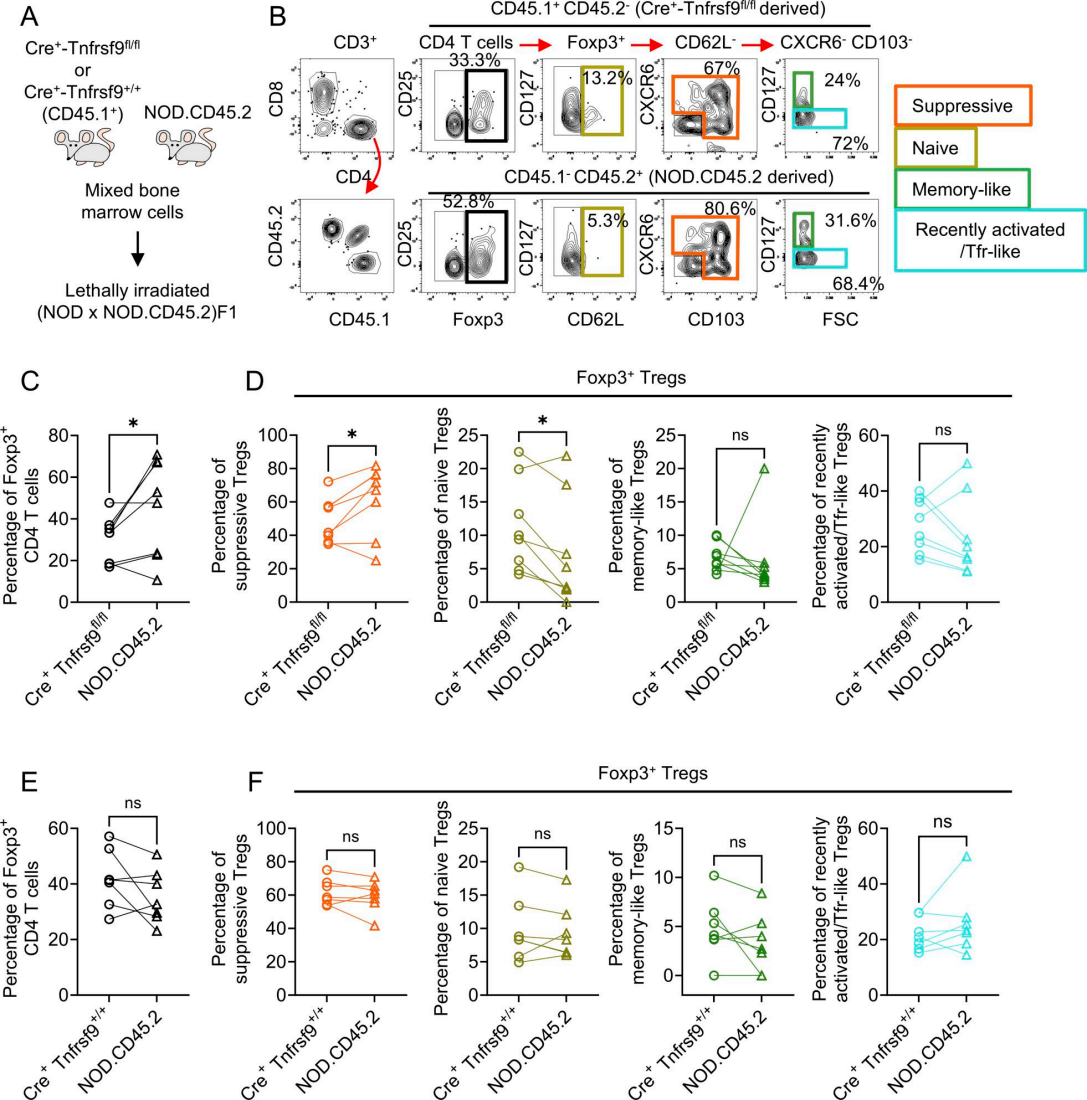
**Differentiation of islet Foxp3** ^**+**^ **Tregs is intrinsically controlled by cell surface CD137. (A)** Design of the MBMC experiment. **(B)** Gating strategy for naïve (CD62L^+^), suppressive (CD62L^−^ CD103^+^ and/or CXCR6^+^), memory-like (CD62L^−^CD103^−^CXCR6^−^CD127^+^), and recently activated/Tfr-like (CD62L^−^CD103^−^CXCR6^−^CD127^−^) subpopulations of Foxp3^+^ Tregs in the islets of the *Cre*^+^-*Tnfrsf9*^fl/fl^ and NOD.*CD45.2* MBMCs. **(C)** Frequencies of *Cre*^+^-*Tnfrsf9*^fl/fl^ and NOD.*CD45.2*-derived Foxp3^+^ Tregs among the respective CD4 T cells in the islets of the MBMCs. *P < 0.05 by a paired *t* test. Results are summarized from three experiments. **(D)** Frequencies of *Cre*^+^-*Tnfrsf9*^fl/fl^ and NOD.*CD45.2*-derived suppressive, naïve, memory-like, and recently activated/Tfr-like subpopulations within the respective Foxp3^+^ Tregs in the islets of the MBMCs. *P < 0.05 by a paired *t* test. ns: not significant. Results are summarized from three experiments. **(E)** Frequencies of *Cre*^+^-*Tnfrsf9*^+/+^ and NOD.*CD45.2*-derived Foxp3^+^ Tregs among the respective CD4 T cells in the islets of the control MBMCs. **(F)** Frequencies of *Cre*^+^-*Tnfrsf9*^+/+^ and NOD.*CD45.2*-derived suppressive, naïve, memory-like, and recently activated/Tfr-like subpopulations within the respective Foxp3^+^ Tregs in the islets of the control MBMCs. ns: not significant by a paired *t* test. Results are summarized from three experiments.

Based on the scRNA-seq results, we designed a flow cytometry antibody panel that identified the four major differentiation states of islet Foxp3^+^ Tregs (naïve, suppressive, memory-like, and recently activated/Tfr-like, respectively, defined as CD62L^+^, CD62L^−^ CD103^+^ and/or CXCR6^+^, CD62L^−^CD103^−^CXCR6^−^CD127^+^, and CD62L^−^CD103^−^CXCR6^−^CD127^−^) (Fig. 7, A and B). The expression patterns of T-bet, TIGIT, LAG3, PD-1, ICOS, CCR8, and CD25 in these four subsets were consistent with the levels of their corresponding transcripts in the scRNA-seq results (Fig. 7, A and B). CD137 was more highly expressed in suppressive and recently activated/Tfr-like subpopulations than naïve and memory-like cells, also reflecting their corresponding *Tnfrsf9* transcript levels (Fig. 7, C and D). Having established the gating strategy for the four islet Foxp3^+^ Treg subpopulations, we next asked whether cell surface CD137 controlled their abundance by comparing *Cre*^+^-*Tnfrsf9*^+/+^, *Cre*^+^-*Tnfrsf9*^E7fl/fl^, and *Cre*^+^-*Tnfrsf9*^fl/fl^ mice. Similar to *Cre*^+^-*Tnfrsf9*^fl/fl^ mice, the frequency of Foxp3^+^ Tregs was significantly reduced in the islets of *Cre*^+^-*Tnfrsf9*^E7fl/fl^ mice (Fig. 7 E). Among islet Foxp3^+^ Tregs, the percentages of naïve cells were significantly higher in *Cre*^+^-*Tnfrsf9*^E7fl/fl^ and *Cre*^+^-*Tnfrsf9*^fl/fl^ than in *Cre*^+^-*Tnfrsf9*^+/+^ mice (Fig. 7 F). Conversely, the frequencies of the suppressive Foxp3^+^ Tregs were significantly lower in *Cre*^+^-*Tnfrsf9*^E7fl/fl^ and *Cre*^+^-*Tnfrsf9*^fl/fl^ than in *Cre*^+^-*Tnfrsf9*^+/+^ mice (Fig. 7 F). These results suggest that cell surface CD137 regulates the differentiation of islet Foxp3^+^ Tregs. Next, mixed bone marrow chimeras (MBMCs) generated with *Cre*^+^-*Tnfrsf9*^fl/fl^ (CD45.1^+^) and NOD.*CD45.2* BM cells were analyzed to further confirm the cell-intrinsic role of CD137 in Foxp3^+^ Tregs (Fig. 8, A and B). As a control, MBMCs were generated using *Cre*^+^-*Tnfrsf9*^+/+^ (CD45.1^+^) and NOD.*CD45.2* BM cells. In islets of MBMCs, the frequency of Foxp3^+^ Tregs was reduced when they could not express CD137 (Fig. 8 C). In addition, Foxp3^+^ Tregs derived from *Cre*^+^-*Tnfrsf9*^fl/fl^ BM had proportionally more naïve and less suppressive subpopulations respectively than the counterparts from the wild-type (Fig. 8 D). In contrast, the differences described above were not observed between *Cre*^+^-*Tnfrsf9*^+/+^ (CD45.1^+^) and NOD.*CD45.2* Foxp3^+^ Tregs in the islets of the control MBMCs (Fig. 8, E and F). Thus, CD137 intrinsically controls the accumulation and differentiation of islet Foxp3^+^ Tregs.

## Discussion

Several TNFRSF members are expressed by Foxp3^+^ Tregs, and studies have identified their unique and overlapping roles in Treg development, differentiation, and functions (Lubrano di Ricco et al., 2020; Mahmud et al., 2014; Vasanthakumar et al., 2017; Watts et al., 2025). Here, we demonstrate that CD137 expression in Foxp3^+^ Tregs plays an important and nonredundant role in restraining ongoing autoimmunity in peripheral tissues. Specifically, complete deletion of CD137 in Foxp3^+^ Tregs leads to significantly increased salivary and lacrimal gland inflammation and substantially accelerated T1D, all of which spontaneously develop in NOD mice. It appears that CD137 expressed by Foxp3^+^ Tregs is not essential for preventing systemic autoimmune inflammation, albeit early T1D onset precludes us from examining older mice to rule out this role. Our study further indicates that both soluble and membrane forms of CD137 contribute to Foxp3^+^ Treg–mediated T1D suppression. In conjunction with previous reports, the current study provides conclusive *in vivo* evidence to show that Foxp3^+^ Treg–derived soluble CD137 is immunosuppressive (Itoh et al., 2019; Kachapati et al., 2012; Kachapati et al., 2013).

The analyses of islet-infiltrating T cells indicate that soluble and cell surface CD137 expressed by Foxp3^+^ Tregs exerted different regulatory functions. The complete absence of CD137 in Foxp3^+^ Tregs promoted effector T cell accumulation, proliferation, and terminal differentiation in islets. Deletion of the cell surface CD137 alone in Foxp3^+^ Tregs did not alter the accumulation and had reduced impacts on proliferation of islet effector T cells, but the latter still displayed a more differentiated phenotype (i.e., increased TIM3 expression in CD8 T cells). Soluble CD137 can directly suppress proliferation and cytokine production by T cells *in vitro* in an APC-independent manner by binding to CD137L expressed on activated T cells (Itoh et al., 2019; Kachapati et al., 2013). In those analyses, CD8 T cells were more affected than CD4 T cells, consistent with the observation in the current study. Soluble CD137 can also suppress T cells by blocking the interaction between CD137L on APCs and CD137 on CD8 T cells, an interaction critical for the diabetogenic activity of CD8 T cells (Foda et al., 2020; Forsberg et al., 2017). Consistently, enhanced expansion and effector differentiation were more prominent for CD8 T cells than Foxp3^−^ CD4 T cells in the lacrimal gland, salivary gland, and islets of Cre^+^-Tnfrsf9^fl/fl^ mice. Collectively, our results suggest that the major target of CD137-dependent Foxp3^+^ Treg–mediated regulation is CD8 T cells. Deletion of cell surface CD137 in Foxp3^+^ Tregs reduced those with a highly suppressive phenotype, as well as their clonal expansion in islets. These highly activated Foxp3^+^ Tregs displayed a gene expression pattern similar to islet CD5^high^ Foxp3^+^ Tregs (Fig. 5 C), which have superior T1D suppressing activity relative to CD5^low^ Foxp3^+^ Tregs (Sprouse et al., 2018). Thus, cell surface CD137 is important for the accumulation of highly suppressive islet Foxp3^+^ Tregs capable of carrying out their regulatory activities through various mechanisms, including tolerogenic interaction with APCs (Dikiy and Rudensky, 2023; Vignali et al., 2008). CD137 on Foxp3^+^ Tregs can also remove CD137L from the cell surface of APCs through trogocytosis (Luu et al., 2021). These collective mechanisms provide an explanation to why complete deletion of CD137 in Foxp3^+^ Tregs had a more profound impact on T1D development than deletion of the cell surface CD137 alone. Human studies have demonstrated that *TNFRSF9* (CD137) is highly expressed in human tumor–infiltrating Tregs across different cancer types, and depletion of CD137^+^ Tregs enhances antitumor responses in mouse models (Buchan et al., 2018; Freeman et al., 2020; Magnuson et al., 2018; Zheng et al., 2021). However, it remains to be determined whether both soluble and cell surface CD137 contribute to the suppressive activity of tumor-infiltrating Tregs.

The current study reveals a CD137-controlled fine balance between CD8 T cells and Foxp3^+^ Tregs. In sharp contrast to the rapid diabetes development observed in *Cre*^+^-*Tnfrsf9*^fl/fl^ mice (CD137 deletion only in Foxp3^+^ Tregs), T1D onset in CD137 or CD137L global knockout NOD mice is delayed (Chen et al., 2014; Foda et al., 2020). CD137 signaling in diabetogenic CD8 T cells is critical for their terminal differentiation, expansion, and survival (Foda et al., 2020; Forsberg et al., 2017). Thus, when CD8 T effectors are compromised, the immunoregulatory role of CD137 in Foxp3^+^ Tregs is masked. This interpretation is further supported by the *Tnfsf9*^−/−^*Cre*^+^-*Tnfrsf9*^fl/fl^ splenocyte transfer experiment. In *Tnfsf9*^−/−^*Cre*^+^-*Tnfrsf9*^fl/fl^ mice where diabetogenic CD8 T cells were impaired, Foxp3^+^ Treg–specific deletion of CD137 did not accelerate T1D development when compared to the wild-type mice. However, upon transfer to CD137L-expressing NOD.*Rag1*^−/−^ recipients where CD137 signaling in CD8 T cells was restored, *Tnfsf9*^−/−^*Cre*^+^-*Tnfrsf9*^fl/fl^ splenocytes caused more rapid diabetes onset than the *Tnfsf9*^−/−^*Cre*^−^-*Tnfrsf9*^fl/fl^ counterparts (Fig. 1 I). CD137 agonistic antibodies have been studied extensively for boosting the antitumor or antivirus activities of CD8 T cells (Salek-Ardakani et al., 2023). Paradoxically, CD137 agonistic antibodies have also been used to suppress autoimmunity in mouse models (Lee et al., 2025; Salek-Ardakani et al., 2023). Our results support that alteration of the balance between CD137^+^ CD8 T cells and CD137^+^ Foxp3^+^ Tregs may improve the efficacy of anti-CD137 antibodies in both disease conditions. Of note, it has been shown that the isotypes of anti-CD137 antibodies dictate their depleting and stimulating activities and an Fc-engineered anti-CD137 antibody capable of depleting Tregs and stimulating CD8 T cells has an enhanced antitumor activity (Buchan et al., 2018).

In contrast to the immunosuppressive role described here, CD137 signaling in Foxp3^+^ Tregs has been shown to cause their dysfunction in skin under chronic Th17-mediated inflammatory conditions (Neuwirth et al., 2025). This study reported that keratinocytes upregulated CD137L in response to a Th17 environment, which in turn stimulated the expression of spermidine/spermine N1-acetyltransferase (SSAT), an enzyme involved in polyamine metabolism, in Foxp3^+^ Tregs and subsequently reduced their suppressive capacity. In our scRNA-seq data, the expression of *Sat1* (encoding SSAT) was not significantly different between *Cre*^+^-*Tnfrsf9*^+/+^ and *Cre*^+^-*Tnfrsf9*^fl/fl^ Tregs (Fig. S4 G), suggesting that wild-type and CD137-deficient Tregs were not differently affected by SSAT in the inflamed islets. Thus, the role of cell surface CD137 in Foxp3^+^ Tregs is context-dependent, possibly influenced by the tissue microenvironment and the disease condition.

Several questions regarding the function of CD137 expressed by Foxp3^+^ Tregs remain to be addressed. Myeloid APCs and B cells are important for T1D development by supporting the activation, differentiation, and expansion of T cells, which in turn stimulate APCs, forming interconnected positive feedback loops to further enhance disease progression (Bass and Bonami, 2024; Ciecko et al., 2025; Zakharov et al., 2020). It remains to be determined whether CD137^+^ Foxp3^+^ Tregs directly suppress the function of myeloid APCs and B cells through soluble or cell surface CD137 to halt T1D progression. Cell surface CD137 is critical for the accumulation and differentiation of Foxp3^+^ Tregs in pancreatic islets of NOD mice, suggesting that local CD137L^+^ APCs sustain activated Foxp3^+^ Tregs in an environment with heightened inflammation. Additional studies are required to identify the APC subsets that support the accumulation of highly activated islet Foxp3^+^ Tregs through the CD137L-CD137 interaction. Currently, we do not know how cell surface CD137 promotes the accumulation of highly activated islet Foxp3^+^ Tregs, albeit scTCR-seq results suggest that CD137 signaling is important for their clonal expansion. Previously, CD137L-containing leukemic extracellular vesicles have been shown to stimulate CD30, TNFR2, and LAG3 expression on Tregs and enhance their suppressive activity *in vitro* (Swatler et al., 2022). In a uropathogenic *Escherichia coli* infection mouse model, CD137L-expressing macrophages promote PD-1 and CTLA4 expression on Foxp3^+^ Tregs (Liu et al., 2025). These studies suggest that CD137L-induced CD137 signaling in Foxp3^+^ Tregs promotes their activation and effector function. CD137 signaling in CD8 T cells has been shown to increase mitochondrial function and fitness (Menk et al., 2018; Teijeira et al., 2018). Proper mitochondrial function is critical for maintaining Foxp3^+^ Tregs and their suppressive activity (Fu et al., 2019; Yang et al., 2017). Together, these results suggest that cell surface CD137 may enhance the accumulation of highly activated islet Foxp3^+^ Tregs by modulating mitochondrial fitness and metabolic programming, a possibility needs to be tested in the future.

In conclusion, our study provides significant insight into the roles of cell surface and soluble CD137 in Foxp3^+^ Tregs. We demonstrate that Foxp3^+^ Tregs are the main producers of soluble CD137 *in vivo* and that both membrane and soluble CD137 expressed by Foxp3^+^ Tregs are important for suppressing autoimmune diabetes through distinct mechanisms. As human Tregs also express cell surface CD137 and produce immunosuppressive soluble CD137, our results highlight the potential for therapeutic modulation of this pathway in human autoimmune diseases.

## Materials and methods

### Mice

NOD/ShiLtJ (RRID:IMSR_JAX:001976), NOD.129S7(B6)-*Rag1*^*tm1Mom*^/J (RRID:IMSR_JAX:003729), NOD.B6-*Ptprc*^*b*^/6908MrkTacJ (RRID:IMSR_JAX:014149), and NOD/ShiLt-Tg(Foxp3-EGFP/cre)1cJbs/J (RRID:IMSR_JAX:008694) were purchased from The Jackson Laboratory (JAX) and maintained at the Medical College of Wisconsin (MCW). The founders of *Tnfrsf9* conditional knockout–ready NOD mice (designated NOD.*Tnfrsf9*^fl/fl^) were generated by JAX Mouse Model Generation Service using the CRISPR/Cas9 system to insert loxP sites flanking the second exon of *Tnfrsf9* directly in NOD embryos. The membrane CD137 conditional knockout–ready NOD mice (designated NOD.*Tnfrsf9*^E7fl/fl^) were generated by JAX Mice Model Generation Service using the CRISPR/Cas9 system to insert loxP sites flanking the seventh exon (encoding the transmembrane domain) of *Tnfrsf9* directly in NOD embryos. Both founders were backcrossed to NOD mice for two generations before intercrossing to fix to homozygosity. NOD.*Tnfrsf9*^fl/fl^ mice were crossed to the NOD.*Foxp3*-*Cre* strain to generate Treg-specific knockout of CD137 (designated *Cre*^+^-*Tnfrsf9*^fl/fl^). Similarly, NOD.*Tnfrsf9*^E7fl/fl^ mice were crossed to the NOD.*Foxp3*-*Cre* strain to generate Treg-specific knockout of membrane CD137 (designated *Cre*^+^-*Tnfrsf9*^E7fl/fl^). *Foxp3*-*Cre* was maintained as a hemizygous state in all Cre-expressing mice. NOD.*Rag1*^*−/−*^.*NY8.3*, NOD.*Tnfsf9*^*−/−*^, NOD.*Tnfrsf9*^*−/−*^, NOD.*Foxp3*-*eGFP*, and NOD.*Tnfrsf9*^−/−^.*Foxp3*-*eGFP* mice have been previously described (Chen et al., 2014; Foda et al., 2020; Forsberg et al., 2017). All mice were used in accordance with and approved by Institutional Animal Care and Use Committee guidelines at the MCW.

### Assessment of T1D and histological analysis

T1D and insulitis development were assessed as previously described (Forsberg et al., 2017). Briefly, T1D development was monitored weekly using urine glucose strips (Diastix; Bayer) with onset defined by two consecutive readings of >250 mg/dl. For histological analysis, all tissues were fixed in a 10% buffered formalin, processed, embedded in paraffin, and sectioned. The pancreas was sectioned at 4 nonoverlapping levels. Granulated β cells were stained with aldehyde fuchsin dye and leukocytes with an H&E counterstain. The insulitis score of each islet was determined as follows: 0—no infiltration; 1—leukocytes surrounding islet but no penetration; 2—estimated loss of up to 25% of the β cells; 3—estimated loss of up to 75% of the β cells; and 4—end stage, <25% of the β cells remaining. When available, at least 30 islets were examined and used to calculate the mean insulitis score for each mouse. For lacrimal and salivary glands, 5-µm sections of paired glands were stained with H&E and inflammation was quantified by light microscopy using standard focus scoring (Barr et al., 2017). Slides were analyzed at 10× magnification to determine the number of mononuclear cell foci in tissue sections, with a focus defined as a cluster of at least 50 mononuclear cells. Slides were scanned using PathScan Enabler IV (Meyer Instruments) to obtain digital images, and tissue areas were measured using ImageJ software (US National Institutes of Health) (Schneider et al., 2012). Focus scores were calculated as the number of foci per 4-mm^2^ tissue area. For skin, lung, liver, kidney, stomach, small intestine, and colon, histological analysis was performed by HistoWiz, Inc. The histology images shown in Fig. S1 B were taken directly from the images of the whole scanned slides using a company-provided web-based tool (https://app.histowiz.com). The scanned slide images have been compressed.

### Flow cytometry analysis

Fluorochrome-labeled antibodies specific for CD3 (RRID:AB_312675), CD4 (RRID:AB_494000), CD8a (RRID:AB_2920943), CD127 (RRID:AB_468793), CD62L (RRID:AB_2925410), CD103 (Cat# 56-1031-82; Thermo Fisher Scientific, RRID:AB_2637111), CD44 (RRID:AB_830785), CD45.1 (RRID:AB_2896116), CD45.2 (RRID:AB_893349), Foxp3 (RRID:AB_465243), CD25 (RRID:AB_2925560), CD19 (RRID:AB_2925618), CD137 (RRID:AB_465864), Ki67 (RRID:AB_2925646), PD-1 (RRID:AB_2734947), ICOS (RRID:AB_2687079), CCR8 (RRID:AB_2629604), T-bet (RRID:AB_925761), CXCR6 (RRID:AB_2721670), TIM3 (RRID:AB_2924459), LAG3 (RRID:AB_11151334), TIGIT (RRID:AB_11042152), SLAMF6 (RRID:AB_2188093), F4/80 (RRID:AB_1548747), and CD11c (RRID:AB_493992) were used. All antibodies were purchased from BD Biosciences, BioLegend, or Thermo Fisher Scientific. Mouse MHC class I (K^d^) tetramers loaded with an islet-specific glucose-6-phosphatase catalytic subunit–related protein mimotope peptide NRP-V7 (KYNKANVFL) were obtained from the National Institutes of Health Tetramer Core Facility (Han et al., 2005). Single-cell suspension was prepared from the spleens, PLNs, thymi, lacrimal glands, salivary glands, and islets of mice at the indicated age. Red blood cells were lysed with the ACK lysis buffer, and then, washed cells were suspended in FACS buffer. Cells were first stained with Zombie NIR (BioLegend) at room temperature for 20 min for assessing cell viability. Cells were then blocked with Fc block (BD Biosciences) at room temperature for 10 min. For tetramer staining, cells were incubated with MHC class I tetramers and Fc block for 15 min at room temperature. Other surface antibodies were then added to stain the cells for 30 min at 4°C. For Treg staining, cells were stained for surface markers, washed with FACS buffer, and then fixed/permeabilized for 30 min using the Foxp3 staining buffer from Thermo Fisher Scientific. Fixed cells were washed twice with permeabilization buffer, stained with anti-Foxp3 antibody for 30 min at 4°C, and then washed twice with permeabilization buffer. Stained cells were resuspended in FACS buffer and acquired using the LSRFortessa ×20 flow cytometer (BD Biosciences) or Cytek Aurora (Cytek) spectral flow cytometer. All flow cytometry data were analyzed with FlowJo software (BD Biosciences).

### Islet isolation and analysis of infiltrating immunocytes

Islet-infiltrating cells were prepared as previously described (Serreze et al., 2005). The pancreas was inflated using a 30-gauge needle by injecting 3–5 ml of HBSS containing 0.5 U/ml collagenase P solution (Roche Diagnostics) and 10 μg/ml DNase (Sigma-Aldrich) into the bile duct. The inflated pancreas was digested at 37°C for 15 min. Next, the digested pancreas was washed three times with 10 ml of HBSS containing 2% fetal bovine serum. Each individual pancreas was then suspended in 5 ml complete RPMI 1640. Islets were visualized under a dissecting microscope and hand-picked. Pelleted islets were suspended in 200 μl of enzyme-free cell dissociation buffer (Life Technologies). Cells were then washed with 500 μl of HBSS, resuspended in FACS buffer, and stained with the indicated antibodies.

### Analysis of soluble CD137

Serum samples were collected from 7- to 9-wk-old *Cre*^+^-*Tnfrsf9*^+/+^, *Cre*^+^-*Tnfrsf9*^fl/fl^, and *Cre*^+^-*Tnfrsf9*^E7fl/fl^ males for analyzing the level of soluble CD137. Splenic T cells were isolated from 7- to 14-wk-old *Cre*^+^-*Tnfrsf9*^+/+^, *Cre*^+^-*Tnfrsf9*^fl/fl^, and *Cre*^+^-*Tnfrsf9*^E7fl/fl^ males using Pan T Cell Isolation Kit II (Miltenyi Biotec) according to the manufacturer’s instructions. Enriched T cells were then stained with anti-CD4, and CD4^+^ Foxp3 (GFP)^+^ Tregs were subsequently purified by FACS on a BD FACSAria II cell sorter. Foxp3^+^ Tregs (2 × 10^5^/well) were seeded in flat-bottom 96-well plates and cultured in the RPMI 1640 medium (Gibco) supplemented with 10% HyClone fetal bovine serum (Cytiva), 1% penicillin–streptomycin (Invitrogen), 55 µM β-mercaptoethanol, 1% MEM nonessential amino acids (Gibco), and 1% GlutaMAX (Gibco). Recombinant mouse IL-2 (20 ng/ml; R&D Systems) was added to the cultures. Cells were incubated for 96 h at 37°C in a humidified incubator with 5% CO_2_. The concentration of soluble CD137 was determined using the mouse 4-1BB/TNFRSF9 DuoSet ELISA kit (R&D Systems) according to the manufacturer’s instructions.

### Adoptive transfer studies

For *in vivo* CD8 T cell proliferation assay, splenocytes were isolated from 7- to 9-wk-old NOD.*Rag1*^*−/−*^.*NY8.3* mice and labeled with 5 mM CFSE (eBioscience) in HBSS at 37°C for 10 min. Cells were then washed with complete RPMI and resuspended in HBSS. The labeled cells (2.5 × 10^7^) were transferred into 7- to 9-wk-old male *Cre*^+^-*Tnfrsf9*^+/+^ and *Cre*^+^-*Tnfrsf9*^fl/fl^ mice. At 4 days after transfer, PLNs were harvested from the recipients for flow cytometry analysis. For T1D incidence study, splenocytes were isolated from 8- to 11-wk-old *Tnfsf9*^−/−^*Cre*^+^-*Tnfrsf9*^fl/fl^ or *Tnfsf9*^−/−^*Cre*^−^-*Tnfrsf9*^fl/fl^ females and 1 × 10^7^ cells were transferred into 5- to 8-wk-old sex-matched NOD.*Rag1*^−/−^ recipients. T1D development was monitored for 20 wk after transfer.

### Generation of MBMCs

BM cells were harvested from the tibias and femurs of 6- to 10-wk-old *Cre*^+^-*Tnfrsf9*^+/+^ (CD45.1^+^), *Cre*^+^-*Tnfrsf9*^fl/fl^ (CD45.1^+^), and NOD.CD45.2 females. T cells were depleted from BMs using anti-CD3e microbeads (Miltenyi Biotec). T cell–depleted NOD.CD45.2 BM cells were mixed with *Cre*^+^-*Tnfrsf9*^+/+^ or *Cre*^+^-*Tnfrsf9*^fl/fl^ BM cells at a 2:3–3:1 ratio, and a total of 5 × 10^6^ BM cells were infused into lethally irradiated (1,100 rads) 5- to 8-wk-old (NOD x NOD.CD45.2)F1 females. BM chimeras were analyzed at 8–9 wk after reconstitution.

### scRNA-seq library preparation and sequencing

For the scRNA-seq experiment, islet-infiltrating cells were isolated from four *Cre*^+^-*Tnfrsf9*^+/+^ and four *Cre*^+^-*Tnfrsf9*^fl/fl^ 7- to 10-wk-old females. Following isolation, cells from two mice of the same strain were pooled (Fig. S3). Next, cell pools were incubated with 5 µg/ml Fc block (anti-mouse CD16/CD32 clone 2.4G2, BioXCell). Each cell pool was then stained with anti-CD45.1, anti-CD4, and unique hashtag oligo (HTO)–conjugated antibodies (BioLegend TotalSeq-C) for 30 min at 4°C, washed, and passed through a 30-µm filter. All stained cells were combined and simultaneously sorted into CD45.1^+^CD4^+^ and CD45.1^+^CD4^−^ samples on a FACSAria III (BD Biosciences). About 12,000 cells were used to prepare individual sequencing libraries using the Chromium Next GEM Single Cell 5′ Reagent Kits version 2 (10X Genomics). Two and three sequencing libraries were respectively prepared for CD4 T cells and non-CD4 T cells. Libraries were quantified using KAPA Library Quantification Kit (Roche) and then sequenced at the University of Wisconsin Biotechnology Center using an Illumina NovaSeq 6000 S2 Reagent kit (100 cycles; Illumina) and NovaSeq Sequencing System (Illumina). Reads were demultiplexed and converted to gene–barcode matrices with mm10 as the reference transcriptome using the Cell Ranger (version 6.0.0) mkfastq and count functions, respectively. HTO-labeled cDNA was amplified separately from gene expression libraries and sequenced. HTO FASTQ files were processed using the Python tool CITE-seq-Count (version 1.4.4) to quantify antibody-derived tags and produce the corresponding feature–barcode count matrix. TCRα/β V(D)J libraries were generated using Chromium Single Cell V(D)J Enrichment Kit and sequenced. Raw FASTQ files were processed using the Cell Ranger V(D)J pipeline (version 6.0.0) to assemble contigs, annotate V, D, J gene usage, and identify productive clonotypes. The raw sequencing data are available at The National Center for Biotechnology Information Gene Expression Omnibus (GSE310947). For scRNA-seq analysis of wild-type and *Tnsrsf*9^−/−^ NOD mice, islet-infiltrating cells were isolated and pooled from six NOD.*Foxp3*-*eGFP* or six NOD.*Tnfrsf9*^−/−^.*Foxp3*-*eGFP* female mice (9–10 wk). Next, cell pools were incubated with 5 µg/ml Fc block (anti-mouse CD16/CD32 clone 2.4G2, BioXCell). Each cell pool was then stained with antibodies against CD45.1, CD4, CD8, CD19, and CD11b for 30 min at 4°C, washed, and passed through a 30-µm filter. CD8 T cells (CD45.1^+^CD8^+^CD4^−^CD19^−^CD11b^−^) and Foxp3^+^ Tregs (CD45.1^+^CD8^−^CD4^+^GFP^+^CD19^−^CD11b^−^) were sorted from each cell pool on a FACSAria III (BD Biosciences). Approximately 7,000 cells from each strain were used for library preparation using Chromium Single Cell 3′ version 2 Reagent Kits (10X Genomics). Libraries were quantified using KAPA Library Quantification Kit (Roche) and then sequenced on an Illumina NextSeq using NextSeq 500/550 High Output Kit version 2 (150 cycles). The raw sequencing data are available at The National Center for Biotechnology Information Gene Expression Omnibus (GSE269611).

### scRNA-seq dataset analyses

Data analysis was performed in R (version 4.3.2) using the package Seurat (version 5.0.1) (Stuart et al., 2019). CD4 T cells and non-CD4 T cells were analyzed independently. All cells from the same genotype were combined for analysis. For quality control, cells with unique feature counts <200 or >5,000 or percentage of counts from mitochondrial genes >20% were removed. Based on hashtag data and function HTODemux, only cells determined as singlet were kept for downstream analysis. From TCR analysis, the resulting filtered_contig_annotations.csv files were imported into R, and information such as TRA, TRB, and CDR3 nucleotide sequences was incorporated with Seurat object as TCR metadata. A clonotype is defined by the paired TCRα and TCRβ chain V and J genes and CDR3 nucleotide sequences. The UMI count data were normalized using the SCTransform method (Hafemeister and Satija, 2019) to adjust for different sequencing depths between cells and variation from percentage of counts from mitochondrial genes. For CD4 T cells in the *Cre*^+^-*Tnfrsf9*^+/+^ and *Cre*^+^-*Tnfrsf9*^fl/fl^ dataset, we restricted the computational analysis to those with successful identification of both TCRα and TCRβ chains to facilitate subsequent combined examination of clonotype and differentiation statuses. Top 3,000 variable genes were selected and followed by principal components analysis to denoise the data in lower dimension. For CD4 T cells, top 10 PCs were used to perform clustering analysis with resolution parameter as 0.3 in the Louvain–Jaccard graph–based algorithm to identify distinctive cell populations. CD4 T cell subsets were determined based on canonical markers and cluster marker genes using the FindAllMarkers function, and the cell differentiation states were assigned according to our previous study (Ciecko et al., 2023). For Treg subclustering, top 10 PCs were used to perform clustering analysis with resolution parameter as 0.3 in the Louvain–Jaccard graph–based algorithm to identify distinctive cell clusters. Their differentiation states were defined based on cluster marker genes and gene module scores, calculated by the AddModuleScore function, using previously defined gene list (Collier et al., 2023; Miragaia et al., 2019; Sprouse et al., 2018). For non-CD4 T cells in the *Cre*^+^-*Tnfrsf9*^+/+^ and *Cre*^+^-*Tnfrsf9*^fl/fl^ dataset, top 20 PCs were used to perform clustering analysis with resolution parameter as 0.1 in the Louvain–Jaccard graph–based algorithm to identify distinctive cell clusters. CD8 T cell clusters were identified based on *Cd3d*, *Cd8a*, and *Cd8b1* expression (Fig. S3). Subsequently, we restricted the computational analysis of CD8 T cells to those with successful identification of both TCRα and TCRβ chains. For CD8 T cell subclustering, top 10 PCs were used to perform clustering analysis with resolution parameter as 0.6 in the Louvain–Jaccard graph–based algorithm. Their differentiation states were defined based on cluster marker genes and gene module scores using previously defined gene list (Collier et al., 2023; Giles et al., 2022). Heatmaps, violin plots, and UMAP plots were generated using DoHeatmap, VlnPlot, and DimPlot functions, respectively. Differential expression analysis between *Cre*^+^-*Tnfrsf9*^+/+^ (wild-type) and *Cre*^+^-*Tnfrsf9*^fl/fl^ (knockout) cluster cells was performed by the FindMarkers function. Pseudotime analysis was performed using Monocle 3 (Cao et al., 2019). To compare CD8 T cells and Foxp3^+^ Tregs from wild-type and CD137 globally deficient NOD mice with the *Cre*^+^-*Tnfrsf9*^+/+^ and *Cre*^+^-*Tnfrsf9*^fl/fl^ cell clusters, we integrated the datasets by performing canonical correlation analysis–based algorithm using the IntegrateData function in Seurat. Specifically, integration anchors were identified between the two datasets using the FindIntegrationAnchors function based on the first 30 PCs and considering five nearest neighbors for anchor identification. These anchors were used to generate a batch-corrected, shared transcriptional space by the IntegrateData function. The cluster identities from the wild-type and CD137 global knockout dataset were projected onto the *Cre*^+^-*Tnfrsf9*^+/+^ and *Cre*^+^-*Tnfrsf9*^fl/fl^ dataset using the TransferData function based on the transfer anchors computed by the FindTransferAnchors function in Seurat.

### Alternative splicing analysis

Wild-type Foxp3^+^ Treg sequencing reads obtained from the library prepared by the Chromium Single Cell 3′ version 2 Reagent Kits (10X Genomics) were used for analyzing *Tnfrsf9* alternative splicing (wild-type cells in Treg-0, Treg-1, Treg-2, and Treg-3 clusters in Fig. 5 D). Raw reads aligned to the *Tnfrsf9* region (chromosome 4:150920155–150946102, mm10) were extracted from the bam files generated by Cell Ranger. Next, we retained only reads that passed default inclusion criteria, such as cell barcode, UMI, and confident transcript assignment, to ensure that only reads contributing to the gene–cell count matrix were analyzed. For each read, we decomposed its alignment into exon-level structure by parsing the CIGAR string, segmenting it into contiguous alignment blocks, which correspond to different genomic regions mapped to the reference genome. These blocks represent either exonic segments or intron-spanning junctions depending on the splicing state of the transcript. By intersecting the genomic coordinate of each block with *Tnfrsf9* exon regions, we identified the specific exons to which each read mapped. After summarizing all UMIs within each cell, we determined the presence or absence of particular exon combinations on a per-cell basis, thereby inferring the targeted alternative splicing state in each cell (e.g., exons 6-7, 6-8, or 7-8 in *Tnfrsf9*). We have developed an R package, exonBlocks, to implement this analysis, and it is available at https://github.com/yuw444/exonBlocks.

### Statistical analysis

Statistical tests were performed in R (version 4.3.2) for scRNA-seq analyses and in GraphPad Prism 10 for other comparisons. An unpaired or a paired *t* test was used for comparison between two groups as indicated. One-way ANOVA followed by Tukey’s multiple comparisons test was used for comparing multiple groups. Adjusted P values were calculated using the Bonferroni correction for multiple comparisons of differentially expressed genes between two groups. A log-rank test was used for comparing T1D incidence between groups. A Wilcoxon signed rank test was used to compare the overall clonotype overlap across T cell clusters.

### Online supplemental material

Fig. S1 describes additional characterization of Cre^+^-*Tnfrsf9*^+/+^ and Cre^+^-*Tnfrsf9*^fl/fl^ mice, including histological analysis of various tissues and flow cytometry analysis of the spleen, PLN, and lacrimal and salivary glands. Fig. S2 describes the frequency and number of IGRP_206-214_-reactive CD8 T cells in the spleen and PLN, as well as the proliferation of adoptively transferred NY8.3 CD8 T cells in the PLN. Fig. S3 describes the study design and cell sorting strategy for scRNA-seq and scTCR-seq analyses, as well as the identification of CD8 T cell clusters. Fig. S4 describes the results from additional analyses of the scRNA-seq and scTCR-seq data, including differential gene expression, clonotype analysis, and *Sat1* expression in CD4 T cell clusters. Fig. S5 describes the results of *Tnfrsf9* alternative splicing and the pseudotime analysis of Treg cluster cells. Table S1 describes CD4 T cell cluster marker genes. Table S2 describes CD4 T cell differentially expressed genes between Cre^+^-*Tnfrsf9*^+/+^ and Cre^+^-*Tnfrsf9*^fl/fl^ mice. Table S3 describes CD8 T cell cluster marker genes. Table S4 describes Treg cluster marker genes.

## Supplementary Material

Table S1 shows CD4 T cell cluster marker genes.

Table S2 shows CD4 T cell wild-type versus KO DE genes.

Table S3 shows CD8 T cell cluster marker genes.

Table S4 shows Treg cluster marker genes.

## Data Availability

Requests for new mouse strains reported here and additional information regarding the study should be directed to and will be fulfilled by the corresponding author. The scRNA-seq and scTCR-seq data generated for this study are available at GEO under the accession number GSE269611. The R package, exonBlocks, generated for this study is available at https://github.com/yuw444/exonBlocks.
